# Soil Application of a Formulated Biocontrol Rhizobacterium, *Pseudomonas chlororaphis* PCL1606, Induces Soil Suppressiveness by Impacting Specific Microbial Communities

**DOI:** 10.3389/fmicb.2020.01874

**Published:** 2020-08-07

**Authors:** Sandra Tienda, Carmen Vida, Ellen Lagendijk, Sandra de Weert, Irene Linares, Jorge González-Fernández, Emilio Guirado, Antonio de Vicente, Francisco M. Cazorla

**Affiliations:** ^1^Departamento de Microbiología, Facultad de Ciencias, Universidad de Málaga, Málaga, Spain; ^2^Instituto de Hortofruticultura Subtropical y Mediterránea “La Mayora”, IHSM-UMA-CSIC, Málaga, Spain; ^3^Koppert Biological Systems, Berkel en Rodenrijs, Netherlands; ^4^Instituto de Hortofruticultura Subtropical y Mediterránea “La Mayora”, IHSM-UMA-CSIC, Estación Experimental “La Mayora”, Algarrobo, Spain

**Keywords:** avocado, *Rosellinia necatrix*, antifungal, biocontrol, soil, rhizosphere, microbial community, suppressiveness

## Abstract

Biocontrol bacteria can be used for plant protection against some plant diseases. *Pseudomonas chlororaphis* PCL1606 (PcPCL1606) is a model bacterium isolated from the avocado rhizosphere with strong antifungal antagonism mediated by the production of 2-hexyl, 5-propil resorcinol (HPR). Additionally, PcPCL1606 has biological control against different soil-borne fungal pathogens, including the causal agent of the white root rot of many woody crops and avocado in the Mediterranean area, *Rosellinia necatrix*. The objective of this study was to assess whether the semicommercial application of PcPCL1606 to soil can potentially affect avocado soil and rhizosphere microbial communities and their activities in natural conditions and under *R. necatrix* infection. To test the putative effects of PcPCL1606 on soil eukaryotic and prokaryotic communities, a formulated PcPCL1606 was prepared and applied to the soil of avocado plants growing in mesocosm experiments, and the communities were analyzed by using 16S/ITS metagenomics. PcPCL1606 survived until the end of the experiments. The effect of PcPCL1606 application on prokaryotic communities in soil and rhizosphere samples from natural soil was not detectable, and very minor changes were observed in eukaryotic communities. In the infested soils, the presence of *R. necatrix* strongly impacted the soil and rhizosphere microbial communities. However, after PcPCL1606 was applied to soil infested with *R. necatrix*, the prokaryotic community reacted by increasing the relative abundance of few families with protective features against fungal soilborne pathogens and organic matter decomposition (*Chitinophagaceae*, *Cytophagaceae*), but no new prokaryotic families were detected. The treatment of PcPCL1606 impacted the fungal profile, which strongly reduced the presence of *R. necatrix* in avocado soil and rhizosphere, minimizing its effect on the rest of the microbial communities. The bacterial treatment of formulated PcPCL1606 on avocado soils infested with *R. necatrix* resulted in biological control of the pathogen. This suppressiveness phenotype was analyzed, and PcPCL1606 has a key role in suppressiveness induction; in addition, this phenotype was strongly dependent on the production of HPR.

## Introduction

Soil is a complex and variable habitats on earth. Soil organisms have developed different mechanisms to survive, function and replicate into a changing environment, with variable moisture, temperature, and chemical contents. Soil conditions can vary in very short distances, but also there is variability over time; therefore, soil organisms must be able to adapt rapidly to different and changing conditions (Thies and Grossman, 2006). Additionally, most of the upper layer of the soils are under the influence of plant roots. Thus, the plant rhizosphere was previously defined as the zone around the root where microorganisms and processes important for plant growth and health are located (Hiltner, 1904). Rhizosphere soil a kind of layer between roots and the surrounding soil, that takes part in the large fluxes of nutrients and non-nutrient compounds (Belnap et al., 2003). Moreover, plant rhizosphere provides a special habitat that promotes higher microbial growth, abundance, and diversity (Praeg et al., 2019).

It is well known that in soil ecosystems, the establishment of plants helps to stabilize microbial community structures and are further modulated after interactions with the plant rhizospheres (Donhauser and Frey, 2018). Plant rhizospheres can be colonized by a high number of microorganisms, reaching cell numbers higher than the number of plant cells, covering between 7% and 15% of the rhizoplane (Gray and Smith, 2005). Plant roots photosynthetically fix carbon, and deposit this carbon directly into their surroundings. These exudates can be used as nutrients by the microbial community, finally influencing their composition and activities (Raaijmakers et al., 2009; Berendsen et al., 2012; Vorholt, 2012).

Soil microorganisms interacts with plant roots and interfere with plant behavior and microbial communities. Many rhizosphere-associated microorganisms can modify seed germination, seedling vigor, plant growth and development, nutrition, diseases, and productivity (Berg et al., 2016). Among them, soilborne plant pathogens are the major limitation in plant production. This group of pathogens is adapted to live in bulk soil; however, the rhizosphere is the place where the pathogen meets the plant and initiates the infection (Raaijmakers et al., 2009). This is also where the complex rhizosphere community of microorganisms can interact with the pathogen and influence the outcome of pathogen infection. Among these microbes, some bacteria positively affect plants and can be considered beneficial bacteria, many of which are designated plant growth-promoting rhizobacteria (PGPR; Lugtenberg and Kamilova, 2009). PGPR can promote beneficial effects on plants. Indirectly, they can inhibit pathogens through competition, colonizing the rhizoplane, inducing plant resistance, and solubilizing minerals, which can cause a modification in the rhizosphere. Directly, PGPR can release antifungal compounds and lytic enzymes (Lugtenberg and Kamilova, 2009).

The increase in knowledge on plant beneficial bacteria has prompted interest in the biological control of plant diseases, which has increased recently because the use of chemicals in the environment provoke public concerns, aiming to the need of finding alternatives to the chemicals used for disease control. Historically, strains with biocontrol potential have been isolated from suppressive soils, studied and used against different soil pathogens (Köhl et al., 2019). Successful, reproducible biological control requires knowledge on the interactions at the root environments, in order to understand the conditions where biocontrol can be obtained (Deacon, 1994; Whipps, 1997) and, indeed, may be part of the reason why more biocontrol agents are reaching the market-place (Whipps and Davies, 2000; Whipps and Lumsden, 2001). The beneficial microorganisms must be mass produced and applied to the crops in a way that optimizes their activities in the corresponding habitat. Microbes can be delivered under different ways, including as liquids (sprays, drenches, and root dips) or as dry formulations applied in-furrow at the time of planting (O’Callaghan, 2016). Several biological control agents (BCAs), composed by living microbial products have been commercialized. A few examples of PGPR developed as commercial products for biological control are *Bacillus subtilis* GBO3 (Kodiak^®^; Gustafson Inc., Dallas, TX, United States), *Pseudomonas fluorescens* A506 (BlightBan^®^; Nufarm Americas, Burr Ridge, IL, United States), *Pseudomonas. aureofaciens* Tx-1 (Spot-Less^®^; Eco Soil Systems Inc., San Diego, CA, United States), *P. syringae* ESC-10, and ESC-11 (Bio-save^®^; Jet Harvest Solutions, Longwood, FL, United States), *Streptomyces griseoviridis* K61 (Mycostop^®^; Verdera Oy, Espoo, Finland), and *S. lydicus* WYEC 108 (Actinovate^®^; Novozymes BioAg Inc. WI, United States) (Figueiredo et al., 2010; Dey et al., 2014).

Among the bacterial biocontrol agents reported, the group of *Pseudomonas* spp. have been extensively studied due to its colonizing ability, inducing plant systemic resistance and to produce antifungal compounds (Haas and Défago, 2005; Weller, 2007). The antifungal compounds produced by rhizospheric *Pseudomonas* spp. are usually the basis for its biological effectiveness and include phenazine-1-carboxylic acid (PCA), phenazine carboxamide (PCN), 2,4-diacetylphloroglucinol (DAPG), pyrrolnitrin (PRN), pyoluteorin (PLT), 2-hexyl 5-propyl resorcinol (HPR), hydrogen cyanide (HCN), siderophores, and some hydrolytic enzymes such as proteases (Ligon et al., 2000; Raaijmakers et al., 2002; Cazorla et al., 2006). Among the antifungal pseudomonads commonly isolated from soil and rhizosphere, *Pseudomonas chlororaphis* is a root-associated bacterial species that displays a wide weaponry of antifungals and shows efficient colonizing abilities (Arrebola et al., 2019; Biessy et al., 2019). Due to these characteristics, commercial or formulated *P. chlororaphis* and related species have been developed to fight soil diseases, especially those due to pathogenic fungi; for example, the commercial product Cerall^®^, composed of the strain *P. chlororaphis* MA342, which shows biological control against phytopathogenic fungi in wheat, rye, and triticale, such as *Fusarium* and *Septoria* (Koppert Biological Systems, Netherlands). Other examples of commercial products based on *P. chlororaphis* and related strains are Cedomon^®^ (*P. chlororaphis* MA 342, BioAgri AB, Sweden), Spot-Less^®^ (*P. aureofaciens* Tx-1, Turf Science Laboratories, Carlsbad, CA, United States) or AtEze^®^ (*P. chlororaphis* 63-28, Turf Science Laboratories, Carlsbad, CA, United States). These formulated *Pseudomonas* spp. have additional activities since they can also be used as bioinsecticides (Kupferschmied et al., 2013) or as rhizobacteria with plant growth-promoting activity (Chen et al., 2015).

Although *Pseudomonas* spp. are widely assayed against different soil diseases, few studies have analyzed the effect of *Pseudomonas* spp. on non-target soil microbial populations. For instance, in barley plants, a transient modification of 3 weeks were observed after *Pseudomonas* spp. DSMZ13134 application (Buddrus-Schiemann et al., 2010). In cucumber, after the application of *P. fluorescens* 2P24 and CPF10, no differences in the bacterial population structure compared to the control were observed after 8 weeks (Yin et al., 2013). On the other hand, in lettuce, the impact of *Pseudomonas jessenii* RU47 on the rhizosphere microbiota was influenced by the soil type 2 or 3 weeks after treatment (Schreiter et al., 2014). Furthermore, all available literature is mainly related to the effect in herbaceous plants, but there is no available information on the effect of *Pseudomonas* application on soil and rhizospheric microbial populations in woody crops.

*Rosellinia necatrix* causes white root rot, a devastating disease in woody plants worldwide (Pliego et al., 2012). Since the 1990s, different studies have established the importance of this disease in avocado (*Persea Americana* Mill.) crops in the Mediterranean area (López-Herrera et al., 1998, 1999). However, the control of avocado white root rot is considered very complex; therefore, several studies have focused on microbial species with the ability to control *R. necatrix* as additional tools to help manage this disease in the future (Cazorla et al., 2006; Ruano-Rosa et al., 2010). Different strategies have been followed to obtain bacterial isolates with biocontrol potential of *R. necatrix. Pseudomonas* spp. and *Bacillus* spp. are commonly isolated from avocado soil and rhizosphere, and some of these strains show antifungal activity and plant protection against soilborne fungal pathogens (Cazorla et al., 2006, 2007; González-Sánchez et al., 2010). Additionally, previous results reported the development of suppressiveness-induced soil after the amendment of commercial soil with composted almond shells in avocado orchards. Induced suppressiveness was directly related to the increase in abundance of specific members of Gammaproteobacteria (including a group of *Pseudomonas* spp. producing antifungal compounds; Vida et al., 2016). These results increased the interest in *P. chlororaphis* strains as potential BCAs against different avocado phytopathogens (Cazorla et al., 2006; Pliego et al., 2008; Arrebola et al., 2019).

In the present study, the model biocontrol rhizobacterium *Pseudomonas chlororaphis* PCL1606 (PcPCL1606) was used. This bacterium showed strong antagonism against various phytopathogenic fungi (including *R. necatrix*) and displayed biocontrol against *Fusarium oxysporum f.* sp. *radicis-lycopersici* and *R. necatrix* (Cazorla et al., 2006; González-Sánchez et al., 2013). The production of the antifungal compound HPR is directly related to the effectiveness of biocontrol and antagonistic activity (Cazorla et al., 2006; Calderón et al., 2013). Furthermore, PcPCL1606 showed additional characteristics related to its fitness in soil and plant roots, such as efficient plant root colonization (González-Sánchez et al., 2010), increased fungal stress symptoms on hyphae after cell-to-cell contact, accelerated hyphal death (Calderón et al., 2014), survival in soil and potential competitiveness with other bacteria associated with the rhizosphere, as PcPCL1606 can produce two recently described bacteriocins (Dorosky et al., 2017).

The objective of this work was to elucidate the impact of PcPCL1606 applications in native communities of soil and rhizosphere, and to unravel the key role of PcPCL1606 applications in soil inducing suppressiveness against *Rosellinia necatrix*.

## Materials and Methods

### Bacterial Strains and Culture Conditions

Rhizospheric *P. chlororaphis* PCL1606 (PcPCL1606; NCBI complete genome accession number GCA_000963835.1) isolated from healthy avocado roots of trees growing in a *R. necatrix* infested area (Cazorla et al., 2006) was used as model pathogen in this study (Table 1). Additionally, a previously obtained Gfp-tagged derivative PcPCL1606 strain (PcPCL1606-GFP, resistant to gentamycin at 80 μg/ml; Calderón et al., 2014) was used as a control to assess survival features (Table 1). To test the role of PcPCL1606 in suppressiveness, a derivative deletion mutant in the *darB* gene (Δ*darB*), impaired in antagonism and biocontrol, was used (Calderón et al., 2019).

**TABLE 1:** Microorganisms and plasmids used in this study.

**Strain**	**Relevant characteristics^a^**	**References**
***Bacterial strains***		
***Pseudomonas chlororaphis***		
PcPCL1606	Wild-type, isolated from Spanish avocado rhizosphere, HPR+, antagonism+, biocontrol+	Cazorla et al., 2006
PcPCL1606-GFP	PcPCL1606 containing the pBAH8 plasmid, expressing the green fluorescent protein (GFP), HPR+, antagonism+, biocontrol+, Gm^r^	Calderón et al., 2014
Δ*darB*	PcPCL1606-derivative deletional mutant in *darB* gene, HPR−, antagonism−, biocontrol−, Km^r^	Calderón et al., 2019
***Fungal strains***		
***Rosellinia necatrix***		
CH53	Wild-type, isolated from avocado white root rot, High virulence	Pérez-Jiménez, 1997
**Plasmids**		
pBAH8	pBBR1MCS-5-containing PA1/04/03-gfp mut3-To-T1; Gm^r^	Huber et al., 2002

*^*a*^HPR: Production of 2-hexyl, 5-propyl resorcinol detected by thin-layer chromatography plates (Cazorla et al., 2006). Antagonism+, fungal antagonism observed in dual plates; biocontrol+, plant protection against *R. necatrix*. Gm^*r*^, resistant to 80 μg/ml of gentamycin; Km^*r*^, resistant to 50 μg/ml of kanamycin.*

Tryptone-peptone-glycerol (TPG; Calderón et al., 2014) medium was used to routinely grow *Pseudomonas* strains at 25°C. Agar–agar (Difco Laboratories, Detroit, MI) was added to a final concentration of 1.5% to produce solid media. The isolates were maintained as glycerol (30%) stocks at −80°C and revived on TPG, and a single colony was used to inoculate each culture.

In this work, a semi-commercial formulated product based on PcPCL1606 was used for experimentation. To obtain a formulated PcPCL1606 product, a standard fermentation of the biologically active product (PcPCL1606) was performed by Koppert B.V. (Berkel en Rodenrijs, Netherlands). Briefly, TPG culture medium was used, and a 3-liter bioreactor was inoculated with a starter culture of PcPCL1606. The culture was grown for 24 h at pH 7.0 and 25°C. The fermentation parameters (oxygen supply and pH) were controlled throughout the fermentation procedure. Antifoam was required during the fermentation process. The fermentation product was harvested 2 to 3 h after the measured oxygen consumption indicated a shift in secondary metabolism (approximately 24 h of growth). Finally, the fermentation product was formulated in a suspension concentrate, comprising cells of the isolate in TPG medium (approximately 5 × 10^9^ colony forming units (cfu)/ml) and was stored at 4°C until its utilization.

For biocontrol and suppressiveness assays, virulent avocado pathogenic *R. necatrix* CH53 was used (López-Herrera and Zea-Bonilla, 2007; Pliego et al., 2009; Table 1). The fungus was grown at 25°C on potato dextrose agar (PDA; Difco Laboratories, Detroit, MI) or TPG plates, and the fungal strain was stored in TPG at 4°C as previously described (Gutiérrez-Barranquero et al., 2012).

### Greenhouse Inoculation Assays: Mesocosm Experiments

**FIGURE 1:**
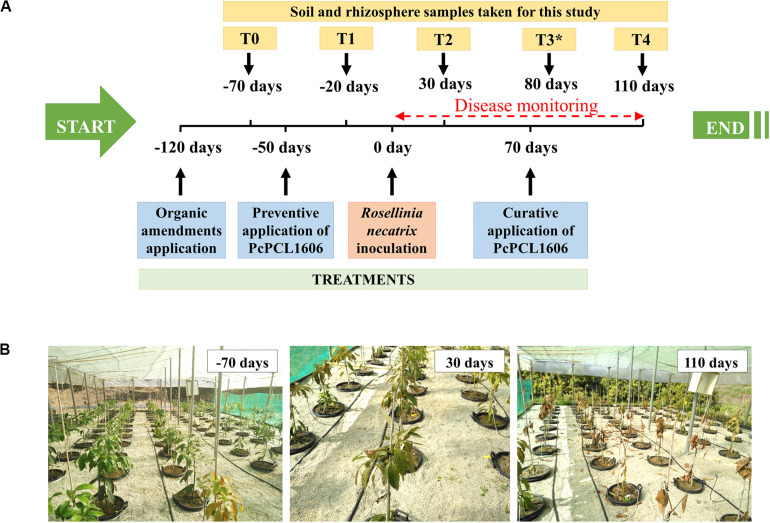
Experimental design of the mesocosm assays. Two independent experiments were performed, treatments in each independent experiment are detailed in Table 2. Seventeen independent mesocosms were used for each of the treatments assayed (eleven mesocosms were inoculated with *R. necatrix*, and six mesocosms remained non-inoculated). **(A)** Schematic view of the mesocosms timeline. Experiments started 120 days (–120 days) prior to the *R. necatrix* inoculation (considered as day 0). One hundred and two 2 years-old avocado plants were sown in commercial soil. At this time started the treatments with the positive control, amending composted almond shells to the corresponding pots. At –50 days, the preventive treatment with PcPCL1606 and PcPCL1606-GFP were applied. 70 days after *R. necatrix* inoculation, the curative PcPCL1606 treatment was applied. Disease monitoring was performed just after *R. necatrix* inoculation. Sampling points for further analysis were stablished at T0 (previous to the preventive treatment), T1 (after preventive treatment), T2 (after *R. necatrix* inoculation), T3* (taken only during biocontrol in “assay 2”) and T4 (end of the experiment). **(B)** Aspect of the mesocosms experiment at –70, 30 and 110 days along the experiment.

Two independent 1-year-long mesocosm experiments were conducted as previously described (Bonilla et al., 2015) to perform disease assessment, bacterial survival and metagenomic analysis. The independent experiments were named “assay 1” (season 2017/18) and “assay 2” (season 2018/19). These assays started 120 days before *R. necatrix* was inoculated (which was considered day 0) and ended 110 days after pathogen inoculation (the experiments last 230 days in total; Figure 1).

Briefly, an experimental microplot platform that mimicked field conditions was designed and constructed for the plant assays at the IHSM-UMA-CSIC “La Mayora” (Algarrobo Costa, Spain, 36°45′37.74″ N – 4°02′26.28″ W). The greenhouse was built as an open structure with double roofing to allow air passage for improved ventilation, and the microplots (mesocosms of 35 liter plant pots) were planted in a white gravel bank to reduce oscillation of the soil temperature. Environmental conditions were monitored during each experiment by using a portable data logger, which recorded the air temperature and relative humidity.

In each independent assay, a total of 102 two-year-old commercial avocado seedling plants (cv. Topa-Topa) were independently transplanted to 35 liter pots filled with a blend (1:1) of solarized natural soil and peat and randomly placed into the experimental area. Each plant into its pot constitute an independent mesocosom. Seventeen independent avocado plants were used for each of the treatments assayed in these studies as listed in Table 2. For each independent treatment, eleven plants were inoculated with *R. necatrix* to study multitrophic interactions during biocontrol, and the remaining six non-inoculated plants were used as controls to study multitrophic interactions without the presence of the pathogen. Fungal inoculation was performed as previously described (Sztejnberg and Madar, 1980; Cazorla et al., 2006). Briefly, four holes per pot were made on the soil surface using a punch, and 16 g of wheat colonized with *R. necatrix* strain CH53 was distributed in the holes before filling them with the surrounding soil.

**TABLE 2:** Main characteristics of the treatments used in the microcosm assay.

**Treatment**	**Assay**	**Assay**	**Code**	**Composition**
	**1**	**2**		
Negative control	✓	✓	Control	No organic amendment and no bacteria were added
Positive control (induced suppressiveness)	✓	✓	ASO	Commercial almond shells derived from almond industry were piled and traditionally composted
Formulated PcPCL1606 preventive	✓	✓	PcPCL1606 preventive	PcPCL1606 formulated in liquid, applied 50 days before inoculating *R. necatrix*
PcPCL1606-GFP preventive	✓	X	PcPCL1606 GFP	PcPCL1606-GFP tagged, applied 50 days before inoculating *R. necatrix*, like the PcPCL1606 preventive treatment
Formulated PcPCL1606 curative	✓	X	PcPCL1606 curative	PcPCL1606 formulated in liquid, applied after the appearance of disease symptoms, 70 days after inoculation with *R. necatrix*

*✓, Included; X, not assayed.*

The first assay, “assay 1,” was designed to test the biocontrol of different treatments with formulated PcPCL1606 as a biologically active product against *R. necatrix* and to study the impact of the application of PcPCL1606 on natural microbial populations with *R. necatrix* inoculation. PcPCL1606 treatments were applied by irrigating a final cell concentration of 1.0 × 10^10^ cfu suspended in 200 ml of sterile water. Treatments were applied using watering to properly distribute the bacterial cells onto the whole pot surface. One of the bacterial treatments was a preventive application (PcPCL1606 preventive), consisting of a single application with a semi-commercial formulate of PcPCL1606 (PcPCL1606 preventive) performed 50 days before inoculation with *R. necatrix*. A second treatment was a curative application (PcPCL1606 curative), using the same semi-commercial formulate of PcPCL1606, which was added after symptoms appeared. The application was performed 70 days after inoculation with *R. necatrix*. A third treatment was included and consisted of a control treatment designed to compare the accuracy of the bacterial counts in different culture media. For this, the PcPCL1606-GFP derivative strain was used in the experiments, following the preventive protocol described above (Table 1 and Figure 1). A bacterial suspension of PcPCL1606-GFP (growing in liquid TPG medium for 24 h at 25°C and 180 rpm) reached a bacterial concentration of 1.4 × 10^9^ cfu/ml. A total of 10^10^ cfu per plant was applied in this treatment (as described above) and allowed specific bacterial counts from soil and rhizosphere samples. In a second assay (“assay 2”), only the preventive semi-commercial formulated PcPCL1606 treatment was repeated to confirm its biocontrol efficacy on the pathogen in the previous assay and to study the impact of PcPCL1606 application on the microbial communities during biocontrol (Table 1 and Figure 1).

In both assay 1 and assay 2, two control treatments were included. First, a positive control of biocontrol consisted of developing soil-induced suppressiveness to *R. necatrix* (Vida et al., 2016). For this, 19 liters of composted almond shells (ASO treatment) was placed on the top layer of 16 liters of soil and peat. This positive control treatment was initiated 150 days before *R. necatrix* inoculation to allow the soil to induce suppressiveness (Figure 1 and Table 1; Vida et al., 2016). The negative control consisted of a group of 17 avocado plants without bacterial treatments.

### Disease Assessment

To study the biological control response of the different treatments, plant disease was monitored weekly for the next 110 days after pathogen inoculation (day 0) in both experiments. Aerial symptoms of white root rot in symptomatic plants was measured using a symptom scale (Bonilla et al., 2015): 0, healthy plant; 1, plant with first symptoms of wilt; 2, overall wilted plant; 3, wilted plant with first symptoms of leaf desiccation; and 4, completely dried plant (dead plant) (see Supplementary Figure S1). The disease index (DI) was calculated for each treatment as previously described (Cazorla et al., 2006). The experiment was considered finished 110 days after inoculation. The area under the disease progress curve (AUDPC) was calculated for statistical comparison of the treatments (Campbell and Madden, 1990) and analized as González-Sánchez et al. (2013).

### Soil and Rhizosphere Sampling

Along the experiment, soil, and rhizosphere samples were taken from each of the assayed treatments at the different sampling points to study the effect of the treatments with formulated PcPCL1606 on the microbial community (Figure 1). During assay 1, soil and rhizosphere samples were taken at different sampling times. Two sampling points were taken before inoculation with *R. necatrix* (considered day 0): one sample was collected before preventive bacterial treatment (T0, −70 days) and a second sample was collected after preventive treatment (T1, −20 days). Samples were collected at two more times after inoculation with *R. necatrix*: at 30 days after treatment (T2) and at the end of the experiment, 110 days after *R. necatrix* inoculation (T4). Following the indications reported in assay 1 and taking into account the biocontrol results from assay 1, in assay 2, soil and rhizosphere sampling was only conducted 80 days after the inoculation with *R. necatrix* (sampling point T3), when the biocontrol was effective after the preventive bacterial treatment with formulated PcPCL1606.

Sampling was performed as described below. Three plants per treatment were randomly selected. Fifteen-centimeter-deep soil core samples were obtained using a 4-cm-diameter core sampler, avoiding to collect close to the inoculation points. Three equidistant points around each plant were sampled and pooled to provide a single composite sample from each plant. Each composite soil and rhizosphere sample per plant was individually processed and analyzed. In the case of the soil and rhizosphere samples taken from three plants challenged with *R. necatrix* in assay 2 at T3, the 3 samples from the untreated control plants were taken from individual symptomatic plants displaying different disease indexes (with disease index 1, 2, or 3; Supplementary Figure S1).

The collected soil and rhizosphere samples were placed in cold storage and transported to the laboratory. From these samples, the roots of avocado, which had adjacent soil, were carefully separated and considered the rhizosphere samples. The rest of the soil was sieved through a 2 mm pore-size sieve. The bulk soil sample was the sieved soil carefully cleared from the roots was considered the bulk soil sample. Fresh soil and rhizosphere samples were used for culture-dependent and culture-independent approaches to perform microbial population analysis. DNA extraction from the soil and rhizosphere samples was also performed immediately after sample collection.

### Microbial Isolation and Plate Counts

Culture-based microbial analysis of Pseudomonas present in the soil and rhizosphere samples was performed. For the soil sample analysis, subsamples of 5 g of the bulk soil were suspended in 40 ml of saline solution (0.85% NaCl) with 5 g of sterile gravel (2 to 4 mm in diameter) and mixed at 250 rpm for 30 min on an orbital shaker, which was followed by 20 min of decantation (Bonilla et al., 2015). For the rhizosphere sample analysis, one gram of the fine roots was homogenized for 2 min in a Stomacher bag with 4 ml of saline solution (Bonilla et al., 2015). The supernatants of both the soil and rhizosphere samples was serially diluted 10-fold; 100 μl of each dilution was plated on different selective media, and bacterial counts were recorded after 48 h at 25°C.

To obtain the bacterial counts of fast-growing heterotrophic bacteria, plates of LB medium amended with cycloheximide (100 mg/liter) were used to prevent fungal growth. To count the number of pseudomonads, the previously described Pseudomonas selective medium (PSM) was used. PSM is composed of King’s B (KB) agar amended with 75 mg of penicillin G, 45 mg of novobiocin, 50 mg of nitrofurantoin and 100 mg of cycloheximide per liter (Larkin and Honeycutt, 2006; Vida et al., 2017).

To study PcPCL1606 survival and to correlate these values with the bacterial count values of PcPCL1606 obtained in PSM with antibiotics, a preventive treatment with PcPCL1606-GFP (gentamicin-resistant strain; Table 1) following an identical protocol as described above was performed during assay 1. Soil and rhizosphere samples were taken along assay 1 to compare the bacterial counts in PSM with those in TPG medium amended with gentamycin (80 mg per liter; TPG-Gm). The same soil and rhizosphere samples were processed as described above and plated on PSM and TPG-Gm medium to compare the bacterial counts after 48 h at 25°C. Typical colony morphology of PcPCL1606-GFP and fluorescence validated the counts of Gm-resistant bacteria.

### DNA Extraction From Soil and Rhizosphere Samples

At each sampling point, DNA was extracted from soil and rhizosphere samples to specifically detect the presence of PcPCL1606 and *R. necatrix*. Additionally, to analyze the effect of PcPCL1606 application on microbial communities of soil and rhizosphere of avocado plants, samples were taken at T2 (80 days after inoculating the bacteria) in assay 1 from the control and preventive treatment where *R. necatrix* had not been applied and analyzed. To test the effect of the preventive PcPCL1606 application during biocontrol, samples from T3 of assay 2 (80 days after inoculating the fungal pathogen) in the control and preventive treatment where *R. necatrix* was inoculated were analyzed. DNA was extracted from each of the 3 independent composite samples where formulated PcPCL1606 was applied (PcPCL1606 preventive) and 3 composite samples where it was not applied (Control). Soil and rhizosphere DNA extractions were performed using 2.0 g of soil or rhizosphere sample and a PowerSoil^®^ DNA Isolation Kit (Qiagen Iberia S.L., Madrid, Spain). The DNA extraction quantity and quality (A260/A230 > 1.8 and A260/A280 > 1.7) were evaluated using a NanoDrop ND-1000 spectrophotometer (NanoDrop Technologies Inc., Wilmington, DE, United States). Additionally, DNA quality was analyzed by agarose gel electrophoresis and RedSafe^TM^ staining (Labotaq, Seville, Spain). DNA was stored at −20°C for further analyses.

### Analysis of 16S rDNA and ITS Gene Sequence

Specific detection of PcPCL1606 in each soil and rhizosphere samples at different sampling times (T0, T1, T2, T3 and T4) was performed by PCR-amplification of a partial sequence inside the gene PCL1606_04860 (with certain homology to the sequence of a glutamine-fructose-6-phosphate aminotransferase from *P. aeruginosa*), which contains a specific 378 nt sequence in PcPCL1606. Specific primers 04860F and 04860R (Supplementary Table S1) were used, and the amplification product was revealed after electrophoresis. Specific detection of *R. necatrix* was performed following previously described procedures (Schena et al., 2002).

To analyze the effect of PcPCL1606 on the microbial communities on the uninoculated samples or during the biocontrol process, metagenomic approaches were followed. For metagenomics analysis, the DNA samples taken from each rhizosphere/soil type were sent to be sequenced by ChunLab (Seoul, South Korea) to obtain the microbial DNA sequences of the 16S rRNA gene and ITS hypervariable regions. For this, a partial16S fragment (428 bp in size) corresponding to the V3-V4 region, was amplified using the PCR primers 341F (CCTACGGGNGGCWGCAG) and 805R (GACTACHVGGGTATCTAATCC), resulting in a 428 bp amplicon (Herlemann et al., 2011). For the ITS amplification, PCR primers ITS1F (CTTGG TCATTTAGAGGAAGTAA; Gardes and Bruns, 1993) and ITS2 (GCTGCGTTCTTCATCGATGC; White et al., 1990) targeted the ITS1F-ITS2 region, resulting in a PCR product of 230 bp in size. PCR products were sequenced by using MiSeq technology (Illumina). Sequences were analyzed using EZbioCloud software (ChunLab)^1^ as follows. Processing raw reads started with quality check and filtering of low quality (<Q25) reads by Trimmomatic version 0.32 (Bolger et al., 2014). After QC pass, paired-end sequence data were merged together using fastq_mergepairs command of VSEARCH version 2.13.4 (Rognes et al., 2016) with default parameters. Primers were then trimmed with the alignment algorithm of Myers and Miller (1988) at a similarity cut off of 0.8. Non-specific amplicons that do not encode 16S rRNA were detected by nhmmer (Wheeler and Eddy, 2013) in HMMER software package version 3.2.1 with hmm profiles. Unique reads were extracted and redundant reads were clustered with the unique reads by derep_fulllength command of VSEARCH (Rognes et al., 2016). The EzBioCloud 16S and ITS rRNA database (Yoon et al., 2017) was used for taxonomic assignment using usearch_global command of VSEARCH (Rognes et al., 2016) followed by more precise pairwise alignment (Myers and Miller, 1988). Chimeric reads were filtered on reads with <97% similarity by reference based chimeric detection using UCHIME algorithm (Edgar et al., 2011) and the non-chimeric 16S and ITS rRNA database from EzBioCloud. After chimeric filtering, reads that are not identified to the species level (with <97% similarity) in the EzBioCloud database were compiled and cluster_fast command (Rognes et al., 2016) was used to perform *de novo* clustering to generate additional OTUs. Finally, OTUs with single reads (singletons) are omitted from further analysis. Finally, the relative abundance of each treatment of eukaryotes/prokaryotes at different taxonomic levels was calculated as the average from three independent samples and was used to perform the comparative distribution analysis.

The secondary analysis, which includes alpha-diversity calculation and biomarker discovery, was conducted by in-house programs of Chunlab, Inc (Seoul, South Korea). All analytics mentioned above were performed in EzBioCloud 16S-ITS based MTP (Microbiome Taxonomic Profile), which is a ChunLab’s bioinformatics cloud platform. The Chao1 index (Chao, 1987) and the Shannon index (Magurran, 2013) were performed as previously described. Rarefaction curves were also applied as previously described (Heck et al., 1975). Beta-diversity was calculated from the relative abundance data (at genus level) from all the samples. The Bray-Curtis index was used to calculate samples similarities. For all analyses, the Fitopac 2.1 software (Shepherd, 2010) was used.

### White Root Rot Suppressiveness Assays

To test *R. necatrix* inhibition by different treated soils, soil suppressiveness assays were performed using a diffusion chamber experiment (Bonilla et al., 2015; Vida et al., 2016). A fungal disk of *R. necatrix* (0.6-cm in diameter) grown on potato dextrose agar (PDA) was placed on a disk of water-agar medium (1% agar; 5 cm in diameter) and transferred to a nitrocellulose filter (pore size 0.45 μm). These systems were placed on soil samples with different treatments taken from assay 2 at sampling point T3. The diffusion chamber was incubated for 5 days at 25°C. The total area of *R. necatrix* growth was measured using Quantity One 1-D analysis software (Bio-Rad Laboratories, Inc., Madrid, Spain) for each soil. Nine replicate chambers per soil type were analyzed. Composted almond shell (ASO)-amended soil was used as suppressiveness positive control. Unamended and untreated controls (Control) and soil where formulated PcPCL1606 had been applied (PcPCL1606 preventive) were assayed.

To verify the role of PcPCL1606 in the suppressiveness of soil samples under the preventive treatment of PcPCL1606, we prepared heat-treated PcPCL1606 preventive soil (HT). Briefly, heat-treated soil consisted of heating the soil in 2 autoclave steps as previously described (Vida et al., 2016). To analyze the restoration of suppressiveness, complemented soil was constructed; HT soil was inoculated with 10^2^ cfu/g soil of PcPCL1606 from a bacterial culture growing overnight (HT + PcPCL1606).

Due to the important role of the antifungal compound HPR in the biocontrol and antagonism of PcPCL1606 (Calderón et al., 2013), supplementation of the HT soil with the Δ*darB* mutant (HT + Δ*darB*) was also performed. Δ*darB* is a mutant deficient in HPR production (not antagonistic and no biocontrol strain) and was used to verify the implication of this compound in suppressiveness (Table 1).

### Data Analyses

Data distributions were tested using one-way analysis of variance (ANOVA) followed by Fisher’s least significant different test with Bonferroni’s correction (*P* = 0.05). All data analyses were performed using IBM SPSS statistics 25 software (SPSS, Inc., Chicago, IL, United States). Based on the standard error, the 95% confidence interval for each response variable was obtained, and the significant differences between the soils were estimated.

## Results

### Biocontrol of Mesocosm Analyses

**FIGURE 2:**
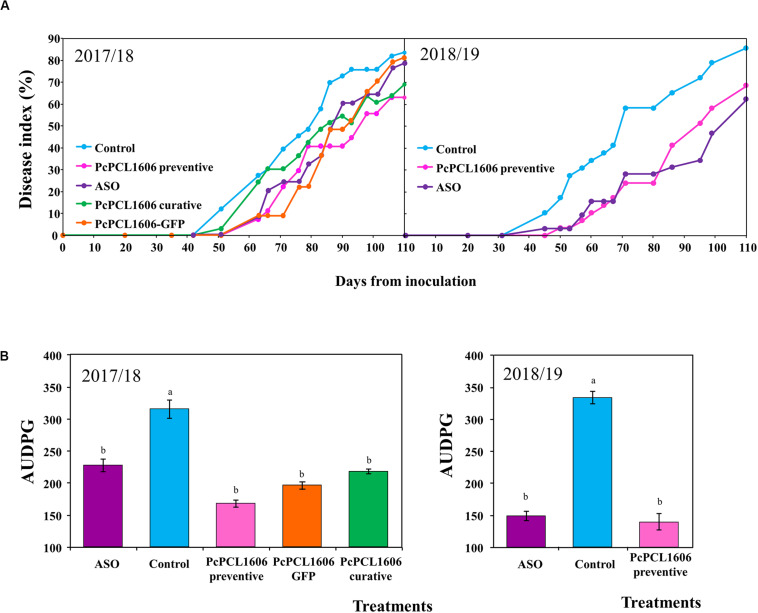
Effect of formulated PcPCL1606 application on avocado white root rot in mesocosm assays. Aerial symptoms of white root rot of the avocado plants under different treatments were calculated, and the time course of the disease index represented in **(A)** assays 1 and assay 2. Area under the disease progress curve (AUDPC) calculated at the end of the **(B)** assay 1 and assay 2. Control, no organic amendment and no bacteria; PcPCL1606 preventive, preventive treatment with formulated PcPCL1606; ASO, composted almond shell; PcPCL1606 curative, curative treatment with formulated PcPCL1606; PcPCL1606-GFP, preventive treatment with the derivative PcPCL1606-GFP. Data were analyzed for significance after arcsine square root transformation with analysis of variance, followed by Fisher’s least significant difference test (*P* = 0.05). Values of bars with different letter indications denote a statistically significant difference.

In mesocosm studies designed to unravel the biocontrol effect of PcPCL1606 application, the first aerial symptoms of white root rot appeared approximately 30 to 40 days after inoculation with *R. necatrix* in both independent microplot assays. The disease index evolution with time is detailed in Figure 2 for each treatment. From the assayed treatments in both assays, the first to show aerial symptoms of white root rot was the unamended control treatment (Control), which reach a disease index above 80%, with many of the inoculated plants already dead at 110 days after *R. necatrix* inoculation.

During assay 1, the positive control of suppressiveness (ASO treatment) showed a delay in symptom appearance but not in symptom reduction. However, during assay 2, ASO treatment induced a delay in appearance and a decrease in white root rot symptoms (Figure 2). In those experiments, the most evident biocontrol effect was produced by the formulated PcPCL1606 treatments. Preventive and curative PcPCL1606 treatments reduced symptom development and reached disease index values of 65 and 70%, respectively (Figure 2). The treatment with PcPCL1606-GFP showed a delay in symptom development but at the end of the experiment reached a disease index level similar to the untreated control, similar to the results of ASO treatment. The temperature and relative humidity data recorded for each experiment showed some differences among seasons, which usually occurs under real field conditions (Supplementary Figure S2). It is important to remark that during assay 1, several days showed unusually low temperatures approximately 60 days after *R. necatrix* inoculation (March–April 2018), coincident with an increase in disease index in some treatments in assay 1. However, a statistical comparison of AUDPC data revealed that all of the assayed PcPCL1606 treatments resulted in a significant reduction (ANOVA, *P* < 0.05) in the disease index when compared to the untreated control plants.

### Effect of PcPCL1606 Applications on Soil and Rhizospheric Culturable Microbial Communities

To count the bacterial levels of PcPCL1606 isolated from soil and rhizosphere samples, the PcPCL1606-GFP strain (Table 1) was used for comparison studies. Bacterial counts from samples under preventive treatment with PcPCL1606-GFP were calculated on TPG-Gm medium to specifically select this bacterial strain and PSM. Plate counts revealed the presence of this strain during the experiment, with levels ranging from 10^3^ to 10^5^ cfu/g and with almost no differences at different sampling times in the rhizospheric and soil samples. A high correlation among the bacterial counts of the two media was obtained, with a regression equation of y = 0.106 + 1.097x (*R* = 0.972) (Supplementary Figure S3).

**FIGURE 3:**
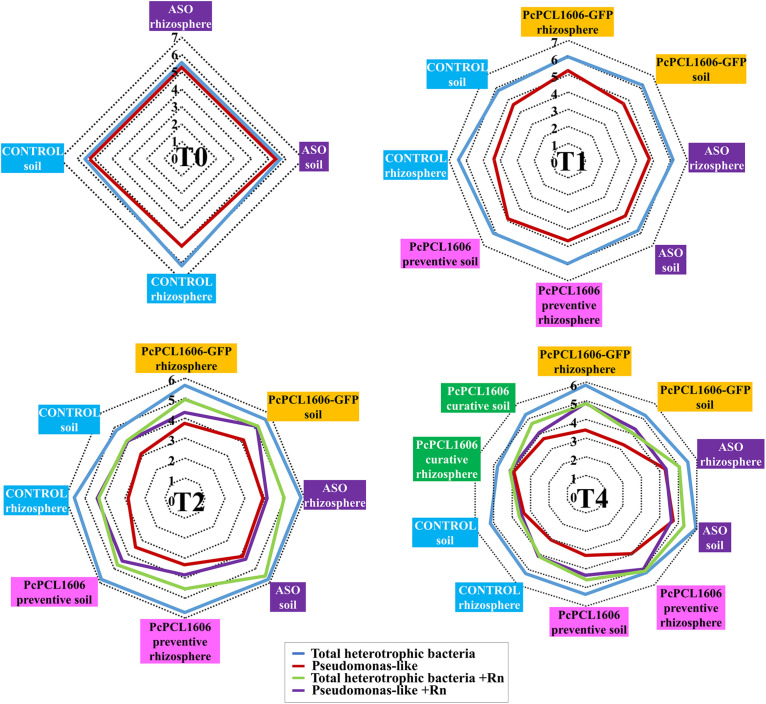
Culturable bacterial populations (total heterotrophic bacteria and pseudomonas-like) during the “assay 1” microcosms experiments. The population densities of fast-growing heterotrophic bacteria and pseudomonas-like were assessed by plate counts at different times (T0, T1, T2, and T4). Bacterial counts obtained from samples taken from inculated plants with *R. necatrix* are noted as +Rn.

Bacterial counts in the bulk soil and rhizosphere samples during assay 1 were performed to analyze the effect of PcPCL1606 treatment on the culturable bacterial populations, especially the group pseudomonas. At T0 (50 days after the initiation of the experiments), bacterial counts were very similar among the samples from the soil and rhizosphere, independent of whether they were taken from the untreated control or the ASO treatments. Bacterial counts of total heterotrophic bacteria were approximately 10^6^ cfu/g of soil or rhizosphere. Pseudomonas-like counts were also approximately 10^6^ cfu/g, with a decrease in count value in rhizosphere samples compared with untreated control plants, with 10^5^ cfu/g of rhizosphere (T0, Figure 3).

Thirty days after PcPCL1606 preventive inoculations (T1, 20 days before inoculation with *R. necatrix*), bacterial counts maintained similar values among soil and rhizosphere samples from the different treatments, with values of total heterotrophic bacteria of approximately 10^6^ cfu/g and slightly lower values for Pseudomonas-like counts, mainly below 10^5^ cfu/g. The samples from untreated control plants had higher total heterotrophic bacterial counts (5 × 10^6^ cfu/g rhizosphere) and, in contrast, showed lower values for Pseudomonas-like counts of approximately 4 × 10^4^ cfu/g of rhizosphere (T1, Figure 3).

In T2 (30 days after inoculation with *R. necatrix*; 80 days after the preventive treatment with formulated PcPCL1606), comparisons among the mesocosm experiments with or without the fungal pathogen were also performed. In general, samples from plants not inoculated with *R. necatrix* showed a slight reduction in total heterotrophic bacterial counts, with higher values of approximately from 7 × 10^5^ cfu/g to 9 × 10^5^ cfu/g in soil and rhizosphere samples from PcPCL1606- and ASO-treated plants (with similar values to those obtained in T1). However, a strong reduction in Pseudomonas-like counts (almost two orders of magnitude less when compared with total heterotrophic bacteria) was also observed, with values ranging from 10^3^ to 10^4^ cfu/g of sample, with lower values detected in samples from the untreated control plants and higher values in the soil and rhizosphere samples from ASO-treated plants (T2, Figure 3). In the plants challenged with *R. necatrix*, lower counts of total heterotrophic bacteria were obtained in general when compared with the unchallenged plants at this same sampling time. Higher values (1–8 × 10^5^ cfu/g) were also displayed by samples from plants under ASO and PcPCL1606-GFP treatments, and lower values were observed with the untreated control plants, with values of approximately from 1 × 10^4^ cfu/g to 3 × 10^4^ cfu/g. On the other hand, in the samples from soil inoculated with *R. necatrix*, the Pseudomonas-like counts were clearly higher when compared with the samples not inoculated with *R. necatrix*. Values ranged from 10^4^ to 10^5^ cfu/g, reaching almost the same value as total heterotrophic bacteria (samples from untreated control plants). In some cases, the Pseudomonas-like levels were not affected by the presence of *R. necatrix*, as shown by the samples taken from the ASO treatment (T2, Figure 3).

Finally, the counts of total heterotrophic bacteria at the end of the experiment (T4; 110 days after *R. necatrix* inoculation; 160 days after preventive treatment with PcPCL1606; 30 days after curative treatment with PcPCL1606), when no *R. necatrix* inoculation was performed, were very similar to those reported in T2, but with a slight reduction in almost all the samples. In these samples, the Pseudomonas-like counts were approximately two orders of magnitude lower than the total heterotrophic bacteria counts. However, the total heterotrophic bacteria counts were higher in some treatments, such as the curative applications of PcPCL1606 (applied 30 days before this sampling point), as well as in the treatment with composted almond shells (ASO) and in the rhizosphere of the preventive treatment with PcPCL1606 (applied 160 before this sampling point) (T4, Figure 3). When the plants were inoculated with *R. necatrix*, bacterial counts from T4 soil and rhizosphere samples showed a similar behavior to T2, with lower levels of total heterotrophic bacterial counts but higher Pseudomonas-like counts. Additionally, in most of the samples, the Pseudomonas-like counts reached similar values as the total heterotrophic bacterial counts. Higher total heterotrophic bacterial counts were detected in ASO samples and in rhizosphere samples preventively treated with PcPCL1606. Interestingly, the Pseudomonas-like counts were also higher in rhizosphere samples preventively treated with PcPCL1606. Remarkably, typical colonies of PcPCL1606-GFP from soil and rhizosphere samples taken from plants that survived after 160 days after inoculation were recovered on TPG-Gm plates with counts above 10^3^ cfu/g.

Because in assay 2, we focused on the confirmation of the biocontrol results previously observed with the PcPCL1606 preventive treatment in assay 1, only one sampling point was taken at T3, when biocontrol was observed. Total heterotrophic bacteria and Pseudomonas-like counts (Supplementary Figure S4) showed values intermediate to those between T2 and T4 in assay 1, ranging from 10^5^ to 10^6^ cfu of total heterotrophic bacteria/g and 10^3^ to 10^4^ cfu of Pseudomonas-like/g.

### Characterization of the Soil Microbial Community Based on 16S rDNA and ITS Sequencing

Sequencing of 16S rDNA and the ITS variable regions elucidated the relative abundances of microbial clades at different taxonomic levels. Comparative distribution analysis were performed only with the most abundant OTUs (≥1% of relative abundance), quantified with a sufficient level of precision due to the high level of OTU richness. In all samples, after sequencing of 16S rDNA, a very low relative abundance of *Archaea* was found (<0.1%).

**FIGURE 4:**
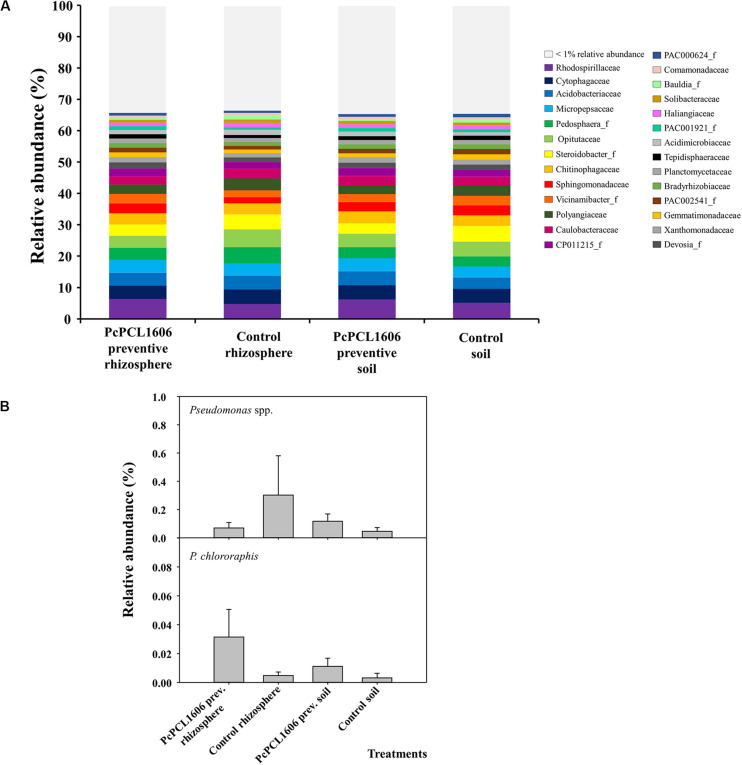
Analysis of prokaryotic communities present in samples of soil and rhizosphere taken from avocado plants non-inoculated with *R. necatrix* during “assay 1” at T2. Negative control of soil (Control soil) and rhizosphere (Control rhizosphere) and samples from formulated PcPCL1606 preventive treatment of soil (PcPCL1606 preventive soil) and rhizosphere (PcPCL1606 preventive rhizosphere) were showed. **(A)** Relative abundance (percentage) of different prokaryotic groups detected by 16S rDNA gene sequencing analysis of soil and rhizosphere DNA at family level. The group with < 1% relative abundance was represented in gray. **(B)** Relative abundance of *Pseudomonas* genus and *Pseudomonas chlororaphis* specie at different treatments.

In assay 1 (at sampling time T2, data available at https://doi.org/10.6084/m9.figshare.12310253.v1), soil and rhizosphere samples from untreated control plants and from plants under preventive treatment with PcPCL1606 and not challenged with *R. necatrix* were analyzed to study the impact of PcPCL1606 treatments on microbial communities. OTUs with a relative abundance above 1% comprised approximately 65% of the relative abundance of prokaryotic microorganisms in soil and rhizosphere samples from plants under the two different treatments (Figure 4). No relevant changes were observed in the relative abundance of prokaryotic families in any of the analyzed samples, with the seven more abundant OTUs (*Rhodospirillaceae*, *Cytophagaceae*, *Acidobacteriaceae*, *Micropepsaceae*, *Pedosphera*_f, *Opitutaceae* and *Steroidobacter*_f) covering approximately 30% of the relative abundance in all analyzed samples (Figure 4A). The 5 most abundant phyla (above 79% of relative abundance) were *Proteobacteria* (42.89%), *Acidobacteria* (10.86%), *Bacteroidetes* (10.42%), *Verrucomicrobia* (8.95%) and *Actinobacteria* (6.04%). At the class level, *Alpha*- and *Gammaproteobacteria* comprised approximately 30% of the relative abundance in the four analyzed samples. Focusing on the relative abundance of members of the *Pseudomonas* genus, the relative abundance was approximately 0.1% in plants under PcPCL1606 treatment and in soil samples of untreated control plants but was 0.3% in rhizosphere samples from untreated plants. The specific relative abundance of *P. chlororaphis* showed levels below 0.01% of the total relative abundance, except in the rhizosphere of PcPCL1606-treated plants, with 3 times more relative abundance (approximately 0.03%; Figure 4B).

**FIGURE 5:**
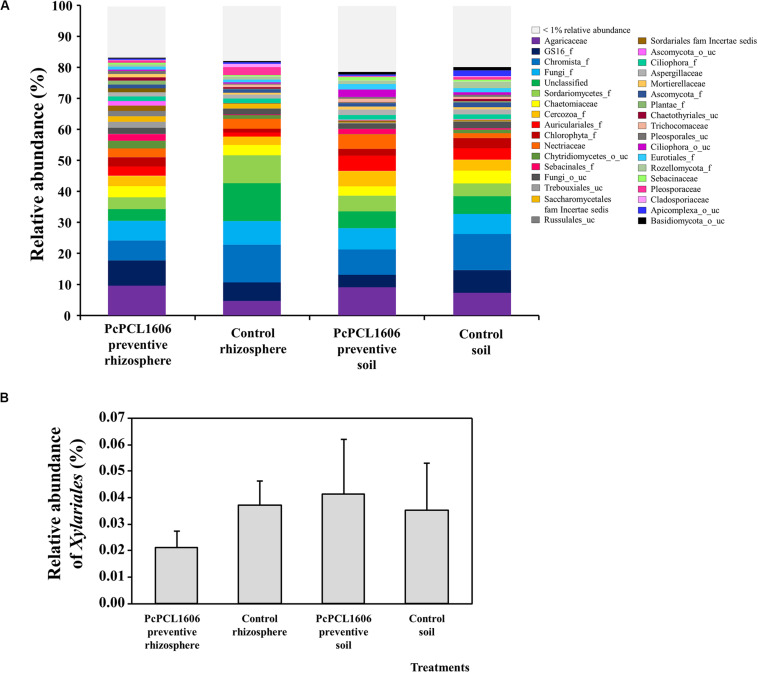
Analysis of eukaryotic communities present in samples of soil and rhizosphere taken from avocado plants non-inoculated with *R. necatrix* during “assay 1” at T2. Negative control of soil (Control soil) and rhizosphere (Control rhizosphere) and samples from formulated PcPCL1606 preventive treatment of soil (PcPCL1606 preventive soil) and rhizosphere (PcPCL1606 preventive rhizosphere) were showed. **(A)** Relative abundance (percentage) of different eukaryotic groups detected by internal transcribed sequences (ITS) sequencing analysis of soil and rhizosphere DNA at family level. The group with < 1% relative abundance was represented in gray. **(B)** Relative abundance of the family *Xylariaceae* at different treatments.

In the same samples, the relative abundance of eukaryotes revealed a similar distribution in the rhizosphere and soil samples treated with PcPCL1606 (Figure 5A). A higher abundance for a family representative of the kingdom of *Chromista* can be observed in samples taken from the untreated control plants. Additionally, in the rhizosphere samples from untreated control plants, a higher relative abundance was observed for an unclassified group and for the class *Sordariomycetes*. Similar results could also be observed at the class level, where the samples from the PcPCL1606 treatment showed a high relative abundance of *Agaricomycetes*, *Sordariomycetes*, *Aphelidiomycetes* and unclassified fungi; however, in the untreated control samples, the relative abundance of *Agaricomycetes* was lower, and the relative abundance of *Chromista* (*Chromista*_g_uc and *Chromista*_c) was higher. In these samples, the relative abundance of *Xylariaceae* was very low (Figure 5B), ranging from 0.02 to 0.04%.

**FIGURE 6:**
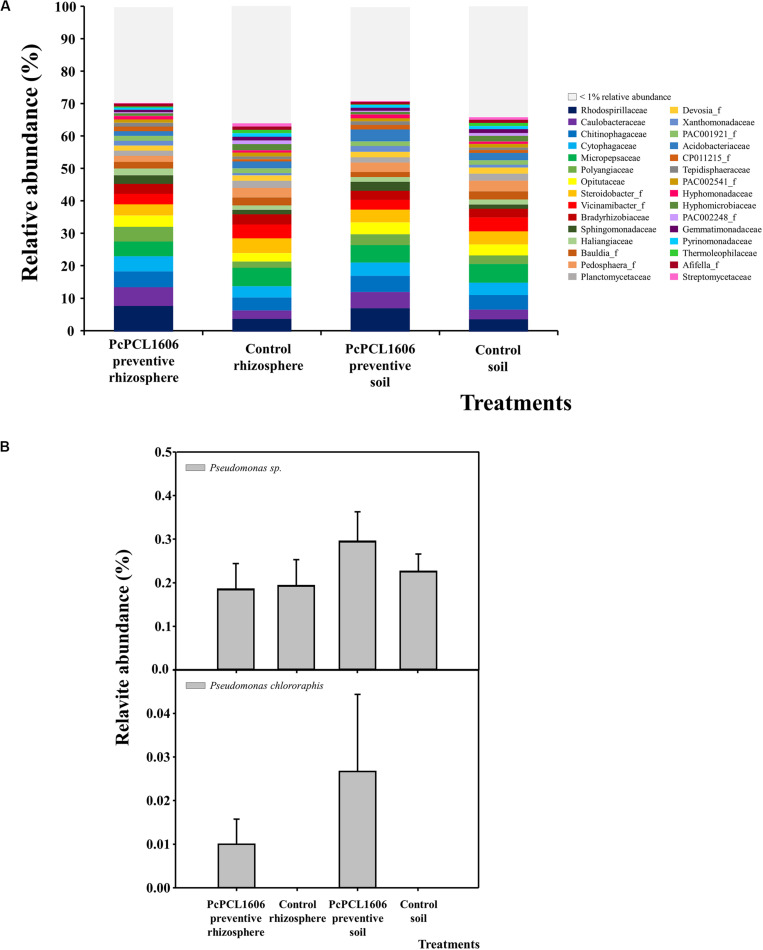
Analysis of prokaryotic communities present in samples of soil and rhizosphere taken from avocado plants inoculated with *R. necatrix* during “assay 2” at T3. Negative control of soil (Control soil) and rhizosphere (Control rhizosphere) and samples from formulated PcPCL1606 preventive treatment of soil (PcPCL1606 preventive soil) and rhizosphere (PcPCL1606 preventive rhizosphere) were showed. **(A)** Relative abundance (percentage) of different prokaryotic groups detected by 16S rDNA gene sequencing analysis of soil and rhizosphere DNA at family level. The group with <1% relative abundance was represented in gray. **(B)** Relative abundance of *Pseudomonas* genus and *Pseudomonas chlororaphis* specie at different treatments.

In soil and rhizosphere samples from microplots artificially inoculated with *R. necatrix*, the analysis of the 16S rRNA sequences (data available at https://doi.org/10.6084/m9.figshare.12309788.v1), revealed a decrease in the relative abundance of the family *Acidobacteriaceae*, but similar groups of microorganisms were found at the family level (Figure 6A) among the different independent experiments (assay 1 and assay 2). Prokaryotic communities from soil and rhizosphere samples treated with PcPCL1606 were almost identical among each treatment and differed from the communities detected in the control samples, which were also more similar among each treatment (Figure 6A). The phylum *Proteobacteria* comprised approximately 50% of the relative abundance, where the class *Alphaproteobacteria* was the most abundant (approximately 30%), followed by the class *Gammaproteobacteria* (ranging from 6.8 to 9.3%). The relative abundance of the genus *Pseudomonas* ranged from 0.2 to 0.3%, with higher values in the soil from samples treated with PcPCL1606 (Figure 6B). Interestingly, the relative abundance of the species *P. chlororaphis* was detectable in the samples from the rhizosphere and soil treated with PcPCL1606 (Figure 6B), but with low values of relative abundance (0.01–0.03%, respectively).

**FIGURE 7:**
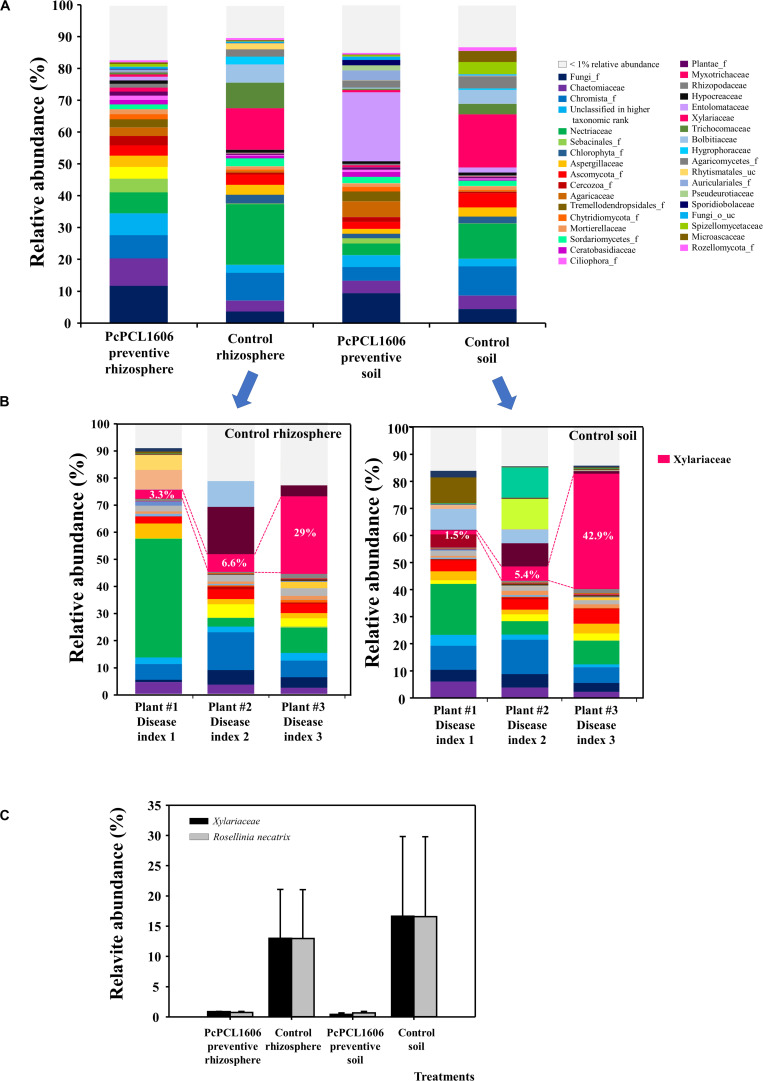
Analysis of eukaryotic communities present in samples of soil and rhizosphere taken from avocado plants inoculated with *R. necatrix* during “assay 2” at T3. Negative control of soil (Control soil) and rhizosphere (Control rhizosphere) and samples from formulated PcPCL1606 preventive treatment of soil (PcPCL1606 preventive soil) and rhizosphere (PcPCL1606 preventive rhizosphere) were showed. **(A)** Relative abundance (percentage) of different eukaryotic groups detected by internal transcribed sequences (ITS) sequencing analysis of soil and rhizosphere DNA at family level. The group with <1% relative abundance was represented in gray. **(B)** Relative abundance of eukaryotic communities on individual samples of soil and rhizosphere from control plants displaying different disease index of white root rot. **(C)** Relative abundance of the family *Xylariaceae* and the specie *R. necatrix* at different treatments.

The ITS sequences were analyzed to reveal the abundance of eukaryotic microbes on the microplots inoculated with the soilborne phytopathogen *R. necatrix*; this allowed us to identify differences in the composition and relative abundance of fungal microbes. A higher number of eukaryotic families with relative abundances equal to or greater than 1% was found in the rhizosphere samples taken from plants under treatments with PcPCL1606, and a lower number was detected in rhizosphere samples from untreated control plants (Figures 7A,B). Eukaryotic communities from samples of rhizosphere treated with PcPCL1606 were very similar among samples, independent of whether they were inoculated or not with *R. necatrix* (Figures 5A, 7A). In general, the introduction of the fungal pathogen *R. necatrix* disturbed the eukaryotic community, leading to different patterns in the different samples and showing a high relative abundance of *Xylariaceae* and *R. necatrix* (Figures 7A,B). To highlight the differences generated by the pathogen, plants displaying different disease indexes (from 1 to 3) were taken, and the soil and rhizosphere of control plants not treated with PcPCL1606 were sampled. The individual analysis of these soil and rhizosphere samples coming from symptomatic plants in the untreated control plants revealed the profound effect of the pathogen, with an increase in the relative abundance of *Xylariaceae* (the family where *R. necatrix* belongs) with an increase in the disease index (Figures 7B,C). However, almost no detection of *Xylariales* or the species *R. necatrix* was observed from the samples treated with PcPCL1606 (Figure 7C). Interestingly, after treatment with PcPCL1606, soil samples showed a high increase in the family *Entolomataceae* (Figure 7A).

**FIGURE 8:**
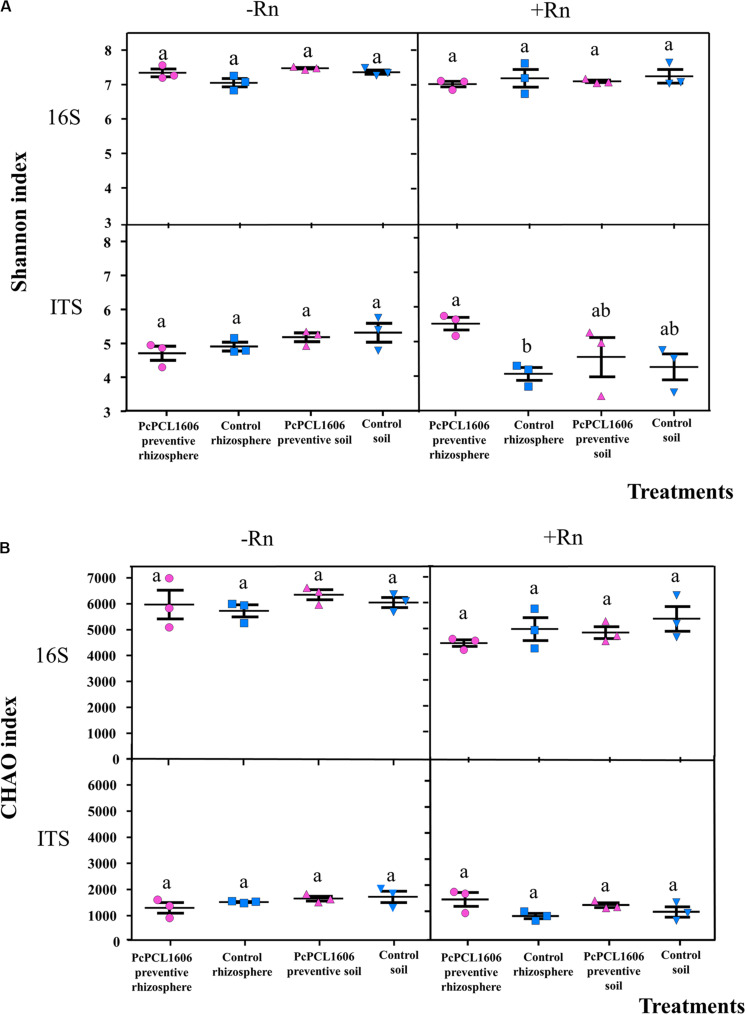
Analysis of diversity with Shannon **(A)** and CHAO **(B)** index of 16S rRNA and ITS sequences from samples of soil/rhizosphere the avocado plant during bicontrol against *R. necatrix*. Samples analyzed were obtained from the negative control of soil (Control soil) and rhizosphere (Control rhizosphere), and samples from formulated PcPCL1606 preventive treatment of soil (PcPCL1606 preventive soil) and rhizosphere (PcPCL1606 preventive rhizosphere). –Rn, uninoculated plants; +Rn, plants inoculated with *R. necatrix.* Values of bars with same letter indications denote a non-statistically significant difference.

The Shannon diversity index and Chao richness index for prokaryotes and eukaryotes in bulk and rhizosphere soils are shown for assay 1 and assay 2, and overall, no differences in microbial diversities between bulk and rhizosphere soil was observed (Figure 8). The Shannon diversity index for prokaryotes was not influenced by the treatment with PcPCL1606, which was independent of whether *R. necatrix* was present (Figure 8). PcPCL1606 preventive application did not significantly influence prokaryotic diversity (Figure 8A). For eukaryotes, no significant differences were observed in non-artificially infected samples; however, a significant difference was found in the Shannon diversity index among rhizosphere samples treated or untreated with PcPCL1606 (Figure 8A). Prokaryotic and eukaryotic richness using the Chao index showed no significant differences among the different samples and treatments (Figure 8B).

Additionally, the Beta-diversity analysis using Bray-Curtis dissimilarities, showed that a preventive application of PcPCL1606 had no influence on the prokaryotic community structure, clustering together with the non-treated samples (Supplementary Figure S5A); however, the presence of *R. necatrix* resulted in a separate clustering of the samples treated or non-treated with PcPCL1606. For the eukaryotic community (Supplementary Figure S5B), the results are very similar, separating the samples with the preventive treatment of PcPCL106 to the samples without bacterial treatment. It is remarkable that eukaryotic communities taken from diseased plants with the lower disease index (samples P1), were allocated in between these two main groups (Supplementary Figure S5B).

### Role of PcPCL1606 in Soil Suppressiveness

**FIGURE 9:**
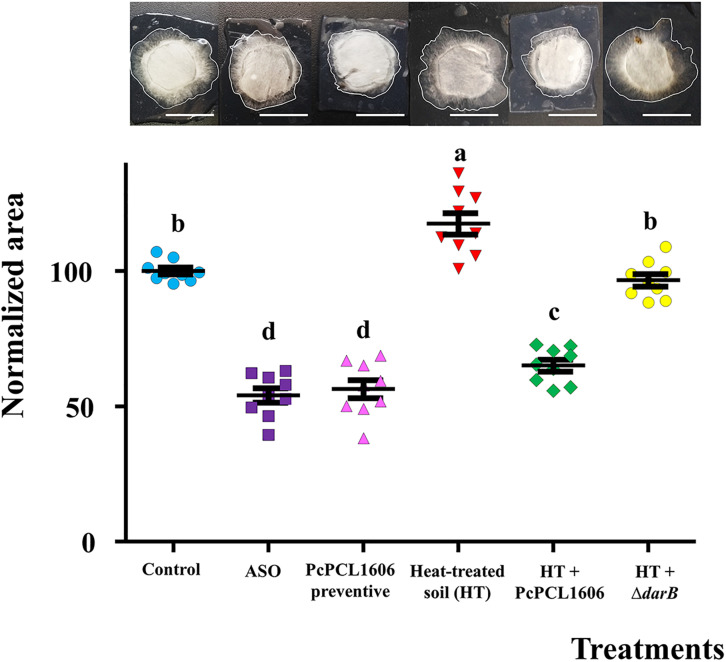
Soil suppressivity assay against *R. necatrix* in diffusion chambers. *R. necatrix* growth area of different soil samples from “assay 2” at T3, used in mesocosm assays. Treatment assayed were Unamended/negative control (Control), composted almond shell (ASO) amended soil was used as positive control of suppressiveness, soil where PcPCL1606 formulated had been applied (PcPCL1606 precentive), heat-treated soil from PcPCL1606 preventive (HT), HT suplemented with PcPCL1606 (HT + PcPCL1606) and HT suplemented with Δ*darB* mutant, non-HPR-producing (HT + Δ*darB*). Values of bars with different letter indications denote a statistically significant difference.

The ability of the different soil samples to inhibit *R. necatrix* was tested using the diffusion chamber assay. The suppressively induced soil after ASO application, as well as combinations with soil treated and untreated with PcPCL1606, were tested. The highest fungal growth inhibition was displayed by the fresh soil amended with composted almond shell and the combinations including a preventive treatment with PcPCL1606, which had a significantly lower area (ANOVA, *P* < 0.05) than the untreated control soil and the rest of the soil combinations (Figure 9). To reveal the microbial nature from suppressiveness, suppressive soil after PcPCL1606 preventive treatment was heat-treated, which abolished its protective phenotype. To assign the protective effect to PcPCL1606, the heat-treated soil was complemented with PcPCL1606, which significantly recovered the suppressiveness. On the other hand, heat-treated soil complemented with the non-antagonistic HPR-defective strain Δ*darB* (Table 1) displayed a clear failure of soil suppressiveness, showing higher fungal colony area of growth (ANOVA, *P* < 0.05) very similar to the growth in heat-treated soil and control soil without any treatment.

## Discussion

*Pseudomonas chlororaphis* PCL1606 (PcPCL1606) has emerged as a potential biocontrol agent in previous works (Pliego et al., 2011). PcPCL1606 has a strong antagonistic and biocontrol activity against several phytopathogenic fungi, among them *R. necatrix* (Cazorla et al., 2006), mainly due to the production of the antifungal compound HPR (Calderón et al., 2013). Previous studies have shown the biocontrol efficacy of PcPCL1606 at different levels (Cazorla et al., 2006; González-Sánchez et al., 2013), and recently, the potential of this strain as a biocontrol agent was also demonstrated in the integrated control against *R. necatrix* in avocado plants; the strain was combined with low concentrations of fungicide, which had a higher plant protection, leading to a reduction in chemical residues and appearance of fungal resistance (Arjona-López et al., 2019). However, one of the final steps to propose PcPCL1606 as a useful and safe biocontrol agent against soilborne fungal pathogens includes the assessment of the presence and abundance of the applied biocontrol agent in soil, testing the efficacy and possible impact on autochthonous soil microbial communities. It is worth noting that this analysis is required previouslu to the registration in Europe of any plant protection products (Commission Regulation No. 544/2011, L155/66).

Survival of inoculated *Pseudomonas* sp. in the soil and plant rhizosphere would be dependent on many factors, such as inoculate formulation, soil conditions, and physiological status of the plant (Wessendorf and Lingens, 1989; van Elsas et al., 1992). In our study, PcPCL1606 was experimentally formulated by Koppert B.V. (Netherlands) and applied by watering the soil around *R. necatrix*-infested plants to mimic the commercial conditions for this treatment. Formulated PcPCL1606 was previously tested in the laboratory and displayed equal antagonism, HPR production, biocontrol and specific amplification by PCR compared with the wild-type strain (data not shown).

It is worth mentioning that PcPCL1606 was previously isolated from avocado roots (Cazorla et al., 2006) in this same area, so it was expected that this bacterium would be very well adapted to this environment and could easily establish its interaction with natural avocado soils and avocado rhizosphere. The PcPCL1606 strain was found in both soil and rhizosphere samples, indicating that this strain can actively move and colonize avocado roots. Survival of PcPCL1606 was confirmed in the soil and rhizosphere at least 160 days after a single inoculation and under environmental conditions. This survival feature has also been shown by other previously reported *Pseudomonas* sp. (e.g., Gao et al., 2012), suggesting that the PcPCL1606 population stabilizes quickly after inoculating into soils and can persist for several months. At the end of the experiment, the survival of PcPCL1606 ranged from 10^3^ to 10^4^ cfu/g, correlating the counts obtained in both media used, TPG-Gm and PSM, indicating that the bacterial counts of PcPCL1606-treated samples in PSM could correspond mainly to the originally formulated PcPCL1606 strain.

It was observed that with only one preventive application (50 days prior to *R. necatrix* inoculation), PcPCL1606 was able to significantly reduce the disease index (25–30%) compared with the untreated plants at the end of two independent experiments, confirming the previously observed biocontrol activity for this strain (Cazorla et al., 2006; González-Sánchez et al., 2013). The results of bacterial counts on the soil and rhizosphere of mesoscosms under different treatments provided a first indication that the presence of *R. necatrix* has an impact on total heterotrophic bacteria and Pseudomonas-like bacterial counts. It was observed that the presence of the fungus stimulated the Pseudomonas-like counts but reduced the total heterotrophic bacteria counts. Similar results have been previously reported for culturable bacterial populations when interacting with other soil fungi. Some bacteria could use fungal-derived substrates and establish different bacteria-fungi relations ranging from mutualistic exudate-consuming to mycophagous interactions (de Boer et al., 2005). This could help to explain the higher survival of PcPCL1606 in the rhizosphere and soil when *R. necatrix* was introduced. Thus, the survival of PcPCL1606 over time would be due to the increase in the available fungal metabolites in the nearby surroundings, which could be easily used by the bacterium. It has been reported that PcPCL1606 is strongly chemotactically attracted by avocado root exudates (Polonio et al., 2017). Once on the root surface, PcPCL1606 can establish microcolonies along the avocado root with the help of exudate compounds in the rhizosphere (Lugtenberg et al., 2001; Calderón et al., 2014). Since PcPCL1606 occupies the same root niches where *R. necatrix* initiates plant infection, the probability that both microorganisms will meet on the avocado root surface and compete for available nutrients is high (Lugtenberg et al., 2001). However, *R. necatrix* also produces exudates that strongly attract PCL1606 (Polonio et al., 2017), finally leading PcPCL1606 to contact the *R. necatrix* hyphae. As a result of such interactions, the bacterial production of antifungal compounds and enzymes would result in a deleterious effect on the fungus, favoring the increase in the Pseudomonas-like counts in the soil and rhizosphere.

The effect of PcPCL1606 applications on the microbial communities when no *R. necatrix* was present was elucidated from natural soil and rhizosphere samples (results from assay 1). In those analyses, no relevant differences in the prokaryotic community structure and composition were observed, independent of whether the samples were taken from rhizosphere or bulk soil or from samples with or without the preventive treatment with PcPCL1606. In these samples, soil and rhizosphere communities were predominated by the phyla *Acidobacteria*, *Actinobacteria*, *Proteobacteria*, *Verrucomicrobia* and *Bacteroidetes*, which are the major phyla observed in soils with moderate inputs of organic matter (Lladó et al., 2017). The classes *Alpha*-, *Beta*- and *Gammaproteobacteria*, and *Acidobacteria* were, in proportion, the dominant bacterial taxa in the avocado soil and rhizosphere samples. These bacterial classes are the most frequently found in high C:N soil (Hermans et al., 2017) and are easily found in soil with the presence of organic matter in decomposition (Johnston et al., 2019). This is the case of the avocado soils of southern Spain, where attempts have been made to increase the low levels of organic matter by leaving leaf litter and chopped pruning waste on the top layer of soil every year (Bonilla et al., 2012) or where organic matter are currently used as amendments (Vida et al., 2016). The effect of PcPCL1606 on eukaryotic communities also revealed minor changes when formulated PcPCL1606 was applied to samples without *R. necatrix*. The clear majority of the eukaryotic communities were composed of fungi, with less than 10% of the eukaryotic ITS sequences belonging to organisms different than fungi (*Cercozoa*_f, *Chlororphyta*_f, *Ciliophora*_f, *Plantae*_f, etc.). It is worthy to note the presence of members from the family *Chromista*_f in all analyzed natural samples. This taxonomic group is widespread (Messenger et al., 2000) but is mainly associated with soils poorly drained, particularly clay soils, were the fundamental factor for the dissemination of spores is water, as observed in the experimental area of this work (Pérez-Jiménez, 2008). Regarding the typical composition of the fungal communities of these samples, members belonging to the saprophytic families commonly associated with organic matter decomposition, such as *Agaricaceae*, *Chytridiomycetes*_f and *Sordariomycetes*_f, were also reported; these families are typically found in environments where leaf litter is decomposed (Kerekes et al., 2013), which occurs with avocado crops. No relevant changes in fungal communities could be observed among the analyzed samples, and the presence of *Xylariaceae* (where the pathogenic fungi *R. necatrix* belongs) was almost not detected, with very low relative abundance. These results were also supported by the absence of significant differences among the diversity and richness indexes. Other studies have reported similar results indicating no relevant changes in natural microbial populations after the application of a biocontrol microorganism. For example, repetitive applications of a soil *P. putida* strain within a citrus orchard showed no effect on the resident microbial community (Steddom et al., 2002) or the treatment with *P. fluorescens* 2P24 and CPF10; after 8 weeks of application in cucumber, the differences in bacterial population structure compared with the control disappeared (Yin et al., 2013). The same have also been observed for Gram-positive biocontrol agents, such as *B. subtilis* B579, which was applied in cucumber plants, with a minimal and transient effect on the rhizosphere bacterial population 4 and 9 weeks after treatment (Chen et al., 2015), and *Bacillus amyloliquefaciens* FZB42, where no taxonomic differences were observed in the rhizosphere microbiota of lettuce 2 and 5 weeks after treatment (Kröber et al., 2014).

When the fungal soilborne pathogen *R. necatrix* was introduced in the mesocosm experiments, the microbial communities of soil and rhizosphere were strongly impacted in different ways. This fungus can attack the plant roots, multiply and expand its hyphae inside the roots, necrotizing the plant living tissues, and finally survive in the decomposing organic matter overwinter (Pliego et al., 2009, 2012). It has been previously described that the presence of a dominant soil fungus can influence the soil and rhizosphere microbial communities (de Boer et al., 2015; Johnston et al., 2019), and in our study, the presence of *R. necatrix* impacted the microbial communities, mainly because they are dependent on the ecological strategy of the dominant fungus (Johnston et al., 2019). The prokaryotic populations of soil and rhizosphere samples showed a slight response to this new biological factor; mainly, the relative abundance of families containing chitinolytic bacteria increased (*Chitinophagaceae* and *Cytophagaceae*). The relative abundance of these groups of chitinolytic bacteria increased in response to the presence of this new and available substrate, resulted by the presence of a soilborne fungal infection (Carrión et al., 2019). The relative abundance of the genus *Pseudomonas* was still low, and under this condition, the species *P. chlororaphis* was only detected in samples taken from plants treated with PcPCL1606, which indicates the strong adaptation of the bacterial biocontrol agent to this specific environment, as previously mentioned.

On the other hand, the eukaryotic community was more impacted by the presence of *R. necatrix*, especially in the taxonomic groups, with relative abundances of approximately 1–2%, which were completely different from samples collected from plants not inoculated. The rhizosphere and soil samples from untreated control plants showed the most evident impact, with 4 taxonomic groups (*Nectriaceae*, *Chromista*-f, *Xylariaceae* and *Trichocomaceae*) responsible for more than 50% of the relative abundance of eukaryotic communities. These observations agree with the obtained results for Beta-diversity, where the main impact onbserved in the analyzed samples was observed in those infested with *R. necatrix*. These impacts on eukaryotic organisms also resulted in a significant difference in the Shannon diversity index for the eukaryotic community, but no differences were observed when analyzing the Chao richness index. The dominant presence of *R. necatrix* in these soils resulted in white root rot disease, which can be considered conducive for this fungus because it shaped the surrounding environment, promoting the appearance of different fungi, mainly saprobes with aggressive colonization strategies and related to the degradation of wood and organic matter (Lasota et al., 2019). In those samples, the relative abundance of *Xylariaceae* and especially the species *R. necatrix* was almost the same in both control soil and rhizosphere samples, with average values above 12%. Since the avocado infection by *R. necatrix* is very aggressive (Pliego et al., 2012), samples to be analyzed were taken from 3 plants showing different disease indexes at the moment of the sampling time. Individual analysis revealed the increase and predominance of *R. necatrix* according to aerial symptoms and the important differences among the relative abundance of fungal families. Although these results were not conclusive, it seems that a succession of different fungi that change in relative abundance takes place during the infection process, with a progressive increase in *R. necatrix*.

However, in these same samples but treated with PcPCL1606, almost no *Xylariaceae* and/or *R. necatrix* were detected (<1%). This preventive PcPCL1606 treatment keeps the microbial communities of the rhizosphere more stabilized resulting in less changes compared with the fungal population from non-inoculated samples treated with PcPCL1606. Notably, the family *Nectriaceae* also increased with the treatment with *R. necatrix*, and several of the species contained in this family are saprobes or weak to virulent, facultative or obligate plant pathogens (Lombard et al., 2015). It is interesting to highlight the increase in relative abundance of the family *Sebacinales* in the rhizospheric and soil samples where formulated PcPCL1606 was applied. *Sebacinales* are highly diverse root symbionts that form various mycorrhizae and endophytic interactions and promote beneficial effects on host plants at diverse levels (Nautiyal et al., 2010; Franken, 2012; Kumar et al., 2012), enhancing abiotic stress resistance (Waller et al., 2005; Baltruschat et al., 2008; Ghimire and Craven, 2011) and resistance to pathogens (Waller et al., 2005; Serfling et al., 2007; Fakhro et al., 2010; Harrach et al., 2013). Interestingly, in soil samples treated with PcPCL1606, a clear impact was observed in fungal communities because the family *Entolomataceae* became predominant (>20% relative abundance). This fungal family is very species-rich, with most of them saprophytic on soil, wood or moss, but some members could be parasitic on plants or even ectomycorrhizal (Co-David et al., 2009). Since no symptoms were observed, these families are more likely to be related to organic matter decomposition or could be in a non-active form.

The suppressiveness displayed by the samples treated with PcPCL1606 can be inferred by the biocontrol experiments, but a more direct analysis showed that the application of PcPCL1606 can confer suppressiveness to the soil 130 days (T3) after the single treatment, similar to the positive control of suppressiveness-induced soil amended with composted almond shells (Vida et al., 2016). This suppressiveness is microbial-based since it completely disappeared after the soil samples were heat treated and can be recovered with a low-dose inoculation of PcPCL1606. Additionally, since HPR was described as the main factor involved in antagonism and biocontrol, soil complementation with the derivative mutant Δ*darB* confirmed the direct involvement of HPR in soil suppressiveness by PcPCL1606.

**FIGURE 10:**
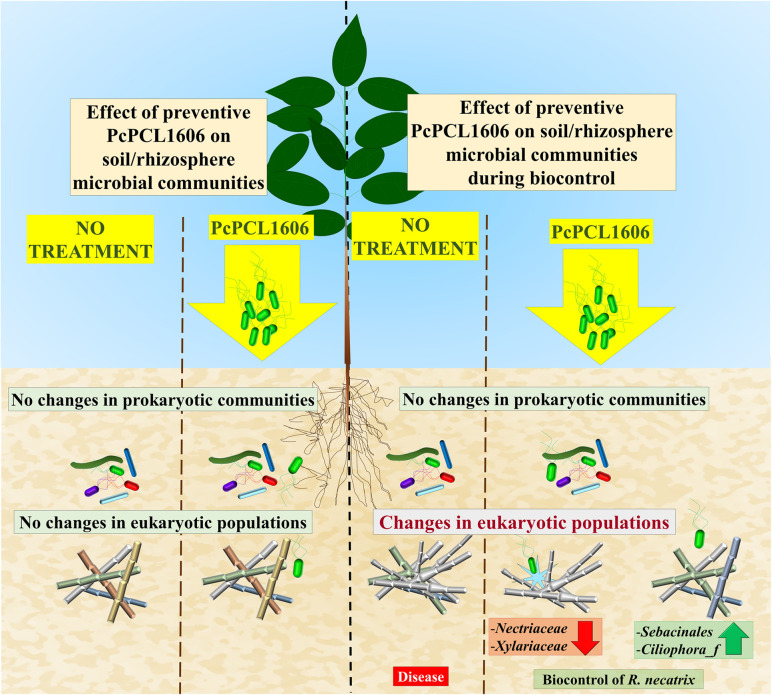
Schematic model for the effect of the preventive application of PcPCL1606. The preventive application of the biocontrol PcPCL1606 strain has no effect on natural prokaryotic and eukariotic populations in the absence of the pathogenic fungus *R. necatrix*. If *R. necatrix* was inoculated in the model, it causes the white root rot disease. The presence of *R. necatrix* disturbed the natural populations and became the predominant fungus. However, when the preventive application of PcPCL1606 take place, resulted in biocontrol of the fungus, reducing its presence and the modification of the microbial communities. A sligh shift in the prokaryotic population was observed, appearing members with potential antifungal activity. And for the eukaryotic communities, reduced the relative abundance of *R. necatrix*. Allowing the development of other fast-growing well adapted fungi likely to be natural competitors.

A conclusion of this work is represented in Figure 10. The application of a formulated PcPCL1606 treatment to the commercial soil of avocado plants did not impact the soil and rhizosphere natural microbial populations (prokaryotic or eukaryotic). However, in a situation under *R. necatrix* infection, the application of PcPCL1606 reduced the symptom development of white root rot disease. Interestingly, the single preventive application allowed us to determine the survival of PcPCL1606 in the soil and avocado rhizosphere for at least 160 days, also conferring biocontrol to the avocado plants against *R. necatrix* infection. During biocontrol, *R. necatrix* impacted the microbial communities; however, that impact was reduced by the preventive application of PcPCL1606. The bacterial populations were poorly influenced by *R. necatrix* introduction and by PcPCL1606 treatment. On the other hand, the severe impact of *R. necatrix* introduction on fungal communities was partially restored by the preventive PcPCL1606 treatment, which inhibited the development of *R. necatrix* and other saprophytic families of fungi that finally led to suppressiveness against this fungus. The basis for this protection is directly related to the production of the compound HPR, which confers the suppressive phenotype to the treated soil.

## Data Availability Statement

The datasets presented in this study can be found in online repositories. The names of the repository/repositories and accession number(s) can be found at: https://doi.org/10.6084/m9.figshare.12310253.v1, 10.6084/m9.figshare.12309788.v1, and https://www.ebi.ac.uk/ena/data/view/ERS4551058-ERS4551081.

## Author Contributions

ST and FC designed the experiments. EL and SW formulated the bacterium. ST, CV, IL, EG, and JG-F performed the experiments. ST, AV, and FC analyzed the results and wrote the manuscript. All the authors read and approved the final manuscript.

## Conflict of Interest

EL and SW were employed by Koppert Biological Systems.

The remaining authors declare that the research was conducted in the absence of any commercial or financial relationships that could be construed as a potential conflict of interest.

## Footnotes

1 www.ezbiocloud.net

## Supplementary Material

FIGURE S1 Disease index of white root rot on 2-years old avocado plants during the microcosms assays. 0, healthy plant; 1, plant with first symptoms of wilt; 2, overall wilted plant; 3, wilted plant with first symptoms of leaf desiccation; and 4, completely dried plant (dead plant).

FIGURE S2 Climatic data during the biocontrol experiments (seasons 2017/18 and 2018/19). Blue bars indicate average relative humidity (HR), and red line indicated average temperature. Data are taken every ten days. Dotted lines indicated season average HR (blue) and temperature (red).

FIGURE S3 Regression analysis of the bacterial counts of PcPCL1606-GFP growing in Pseudomonas selective medium and in TPG amended with gentamicin.

FIGURE S4 Effect of formulated PCL1606 application on culturable microbial populations during the biocontrol, taken at T3 during the “assay 2” microcosms experiments. The population densities of fast-growing heterotrophic bacteria and pseudomonads-like were assessed by plate counts at different times (T0, T1, T2, and T3). Bacterial counts from samples inoculated with *R. necatrix* were showed as +Rn.

FIGURE S5 Analysis of structure using the Bray-Curtis index of 16S rRNA **(A)** and ITS **(B)** sequences from samples of soil/rhizosphere the avocado plant during bicontrol against *R. necatrix*. Samples analyzed were obtained from the negative control of soil (Control soil) and rhizosphere (Control rhizosphere), and samples from formulated PcPCL1606 preventive treatment of soil (PcPCL1606 preventive soil) and rhizosphere (PcPCL1606 preventive rhizosphere). +Rn: plants inoculated with *R. necatrix*.

TABLE S1 Specific primers for specific amplification of *Pseudomonas chlororaphis* PCL1606 (PcPCL1606) and *R. necatrix* from soil and rhizosphere DNA.
