# Supplementary figures and images for: Soil Application of a Formulated Biocontrol Rhizobacterium, Pseudomonas chlororaphis PCL1606, Induces Soil Suppressiveness by Impacting Specific Microbial Communities
Source: Front Microbiol. 2020 Aug 7;11:1874. doi: 10.3389/fmicb.2020.01874 (PMC7426498; doi:10.3389/fmicb.2020.01874)

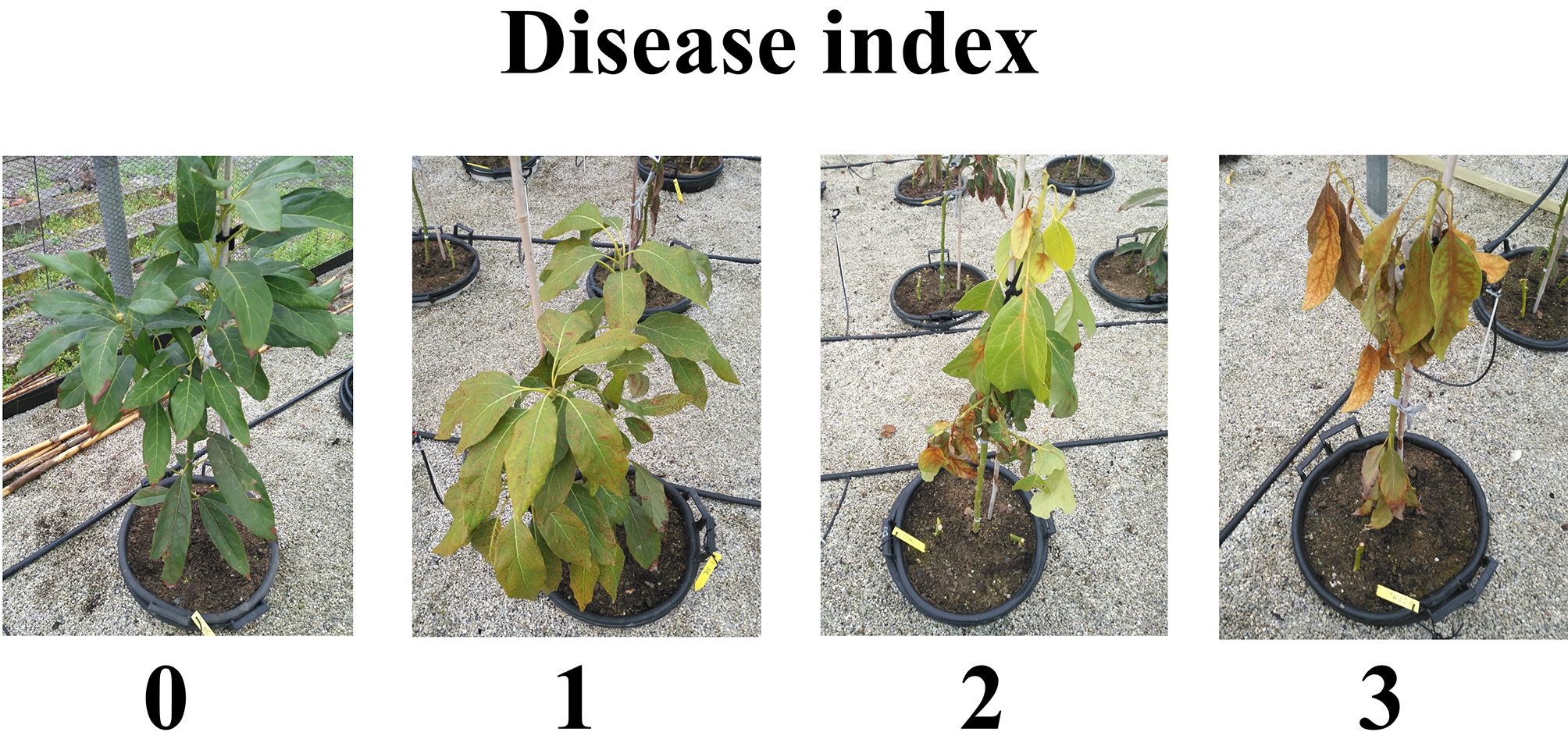

Supplement: FIGURE S1 — Disease index of white root rot on 2-years old avocado plants during the microcosms assays. 0, healthy plant; 1, plant with first symptoms of wilt; 2, overall wilted plant; 3, wilted plant with first symptoms of leaf desiccation; and 4, completely dried plant (dead plant). [file Image_1.TIF]

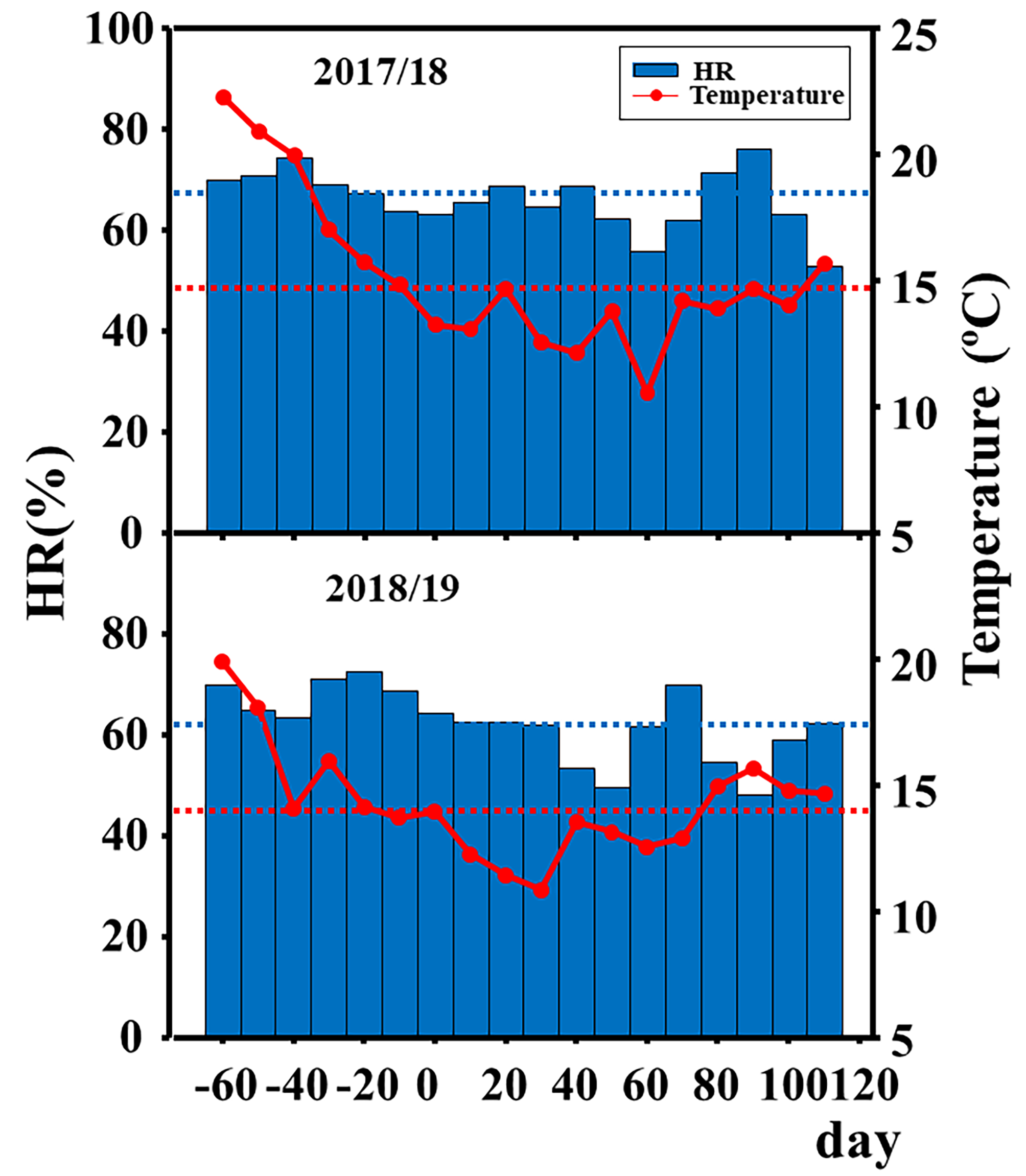

Supplement: FIGURE S2 — Climatic data during the biocontrol experiments (seasons 2017/18 and 2018/19). Blue bars indicate average relative humidity (HR), and red line indicated average temperature. Data are taken every ten days. Dotted lines indicated season average HR (blue) and temperature (red). [file Image_2.TIF]

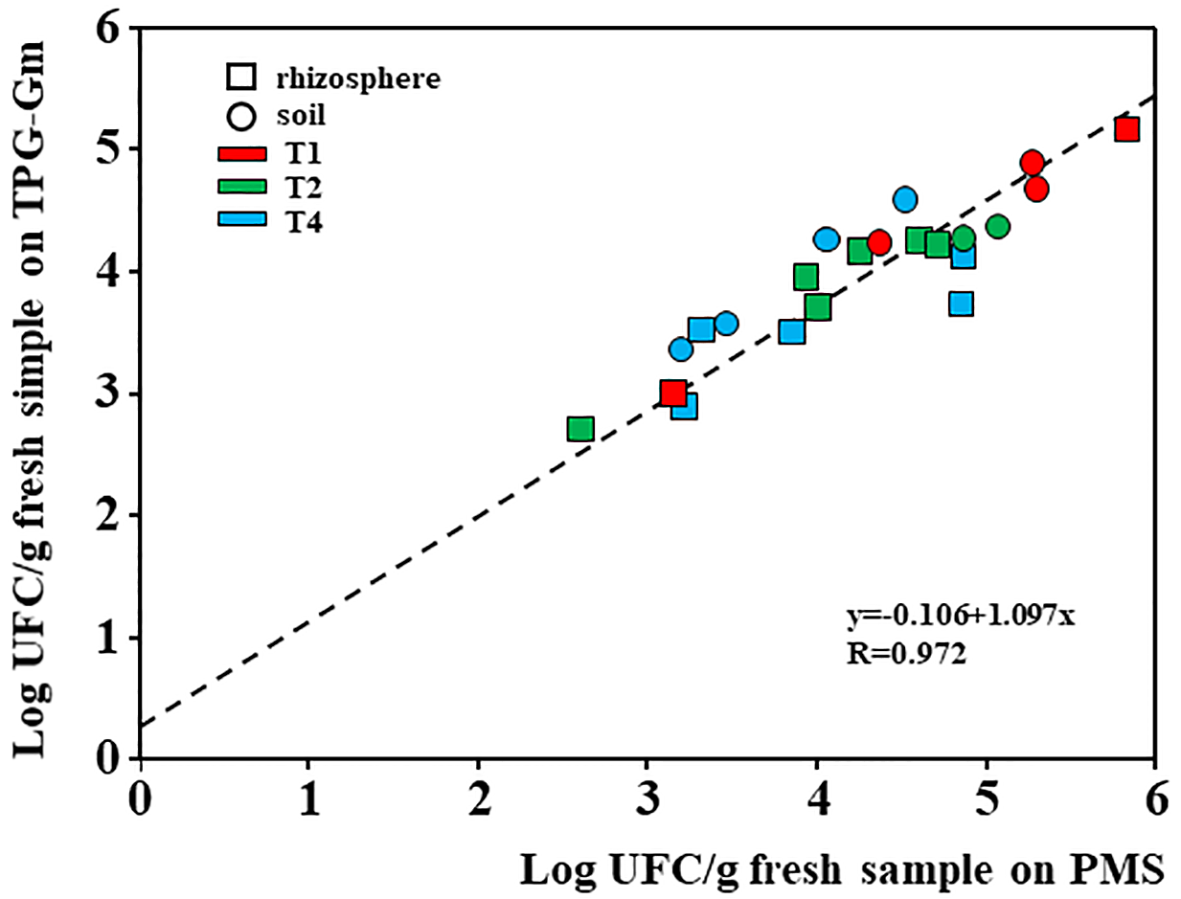

Supplement: FIGURE S3 — Regression analysis of the bacterial counts of PcPCL1606-GFP growing in Pseudomonas selective medium and in TPG amended with gentamicin. [file Image_3.TIF]

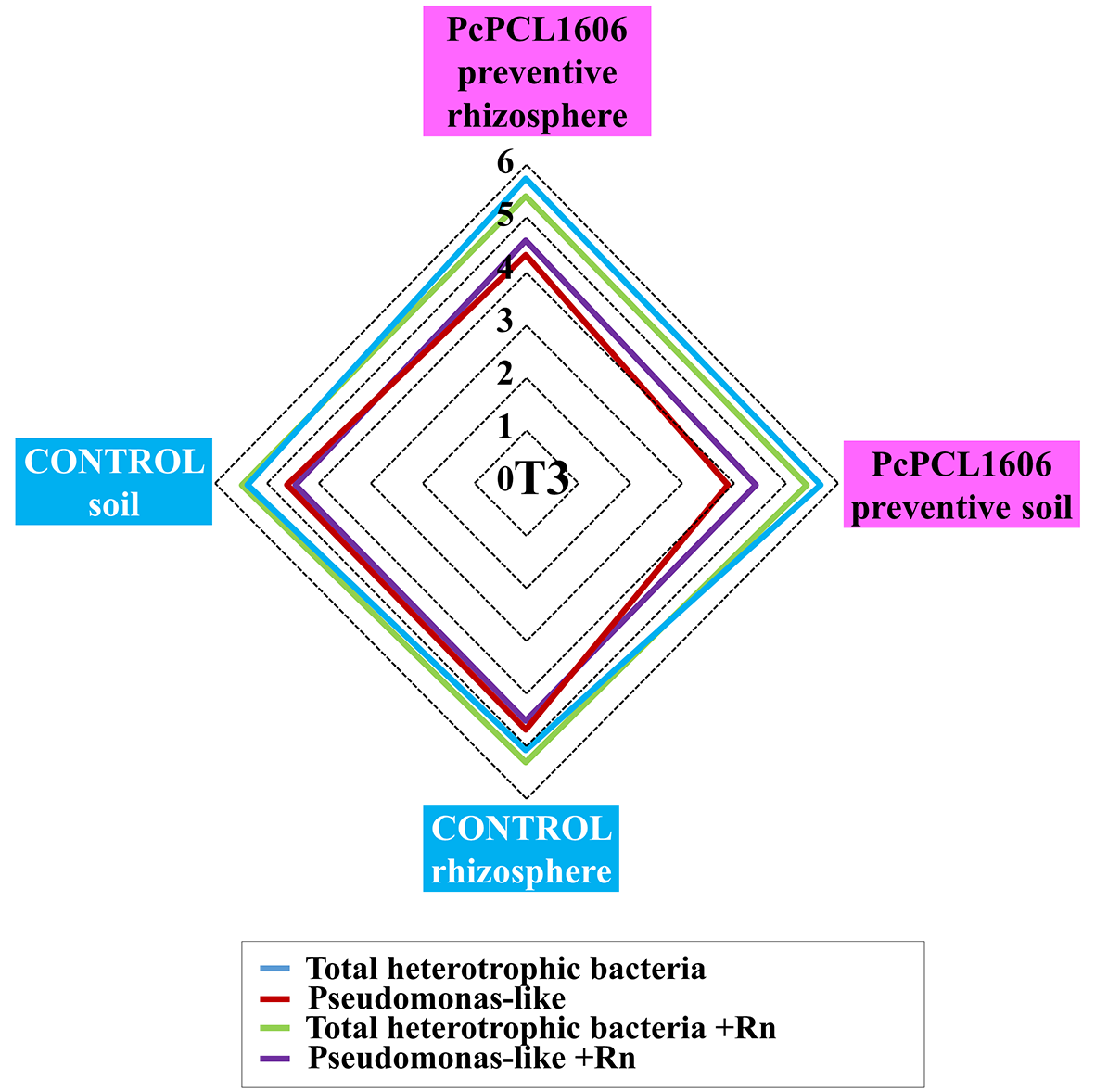

Supplement: FIGURE S4 — Effect of formulated PCL1606 application on culturable microbial populations during the biocontrol, taken at T3 during the “assay 2” microcosms experiments. The population densities of fast-growing heterotrophic bacteria and pseudomonads-like were assessed by plate counts at different times (T0, T1, T2, and T3). Bacterial counts from samples inoculated with R. necatrix were showed as +Rn. [file Image_4.TIF]

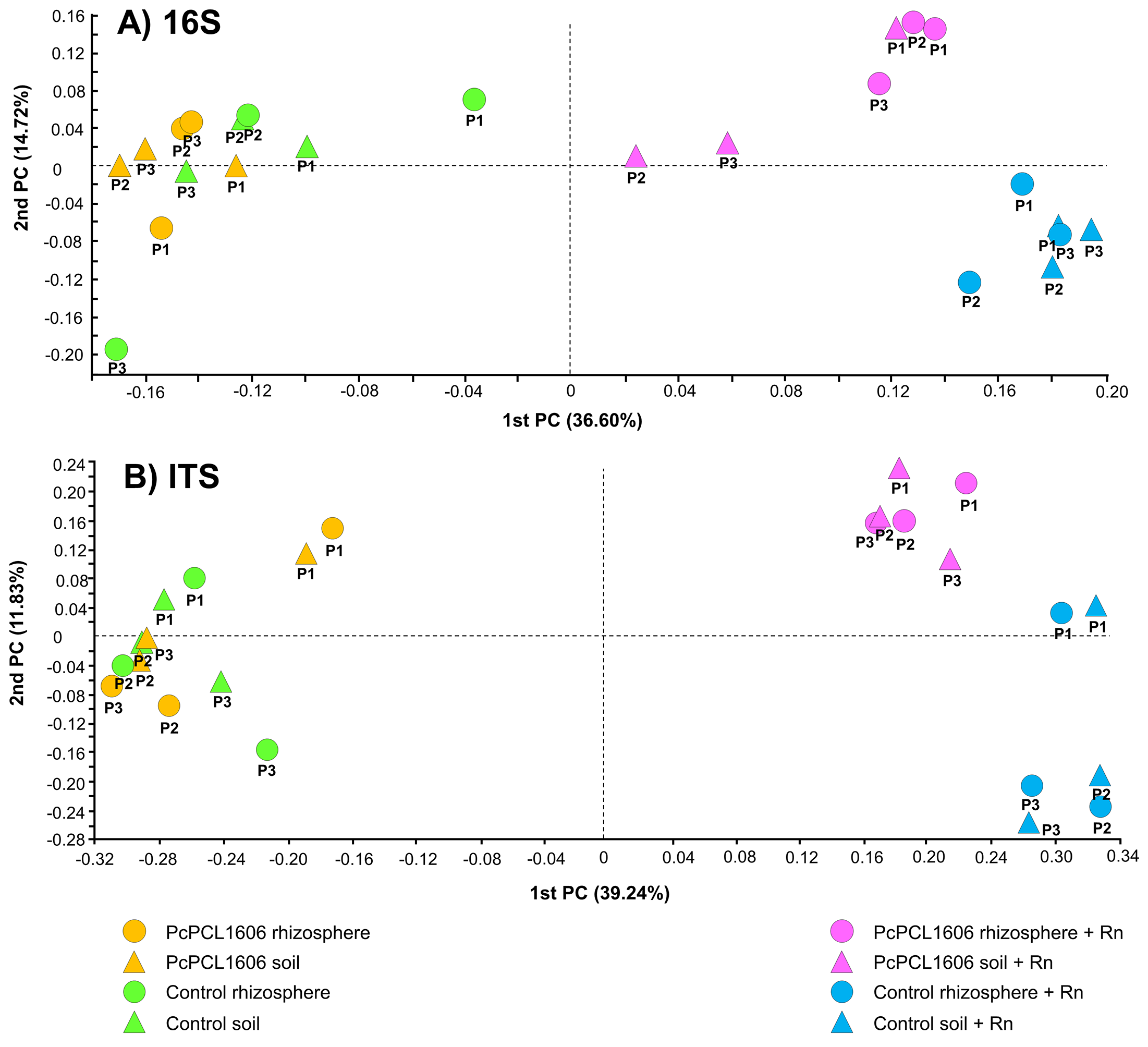

Supplement: FIGURE S5 — Analysis of structure using the Bray-Curtis index of 16S rRNA (A) and ITS (B) sequences from samples of soil/rhizosphere the avocado plant during bicontrol against R. necatrix. Samples analyzed were obtained from the negative control of soil (Control soil) and rhizosphere (Control rhizosphere), and samples from formulated PcPCL1606 preventive treatment of soil (PcPCL1606 preventive soil) and rhizosphere (PcPCL1606 preventive rhizosphere). +Rn: plants inoculated with R. necatrix. [file Image_5.TIF]
