# Supplementary material for: Soil Application of a Formulated Biocontrol Rhizobacterium, Pseudomonas chlororaphis PCL1606, Induces Soil Suppressiveness by Impacting Specific Microbial Communities
Source: Front Microbiol. 2020 Aug 7;11:1874. doi: 10.3389/fmicb.2020.01874 (PMC7426498; doi:10.3389/fmicb.2020.01874)
Supplement: TABLE S1 — Specific primers for specific amplification of Pseudomonas chlororaphis PCL1606 (PcPCL1606) and R. necatrix from soil and rhizosphere DNA. [file Table_1.DOCX]

Table S1: Specific primers for specific amplification of *Pseudomonas chlororaphis* PCL1606 (PcPCL1606) and *R. necatrix* from soil and rhizosphere DNA

| **Specific PCR for** | **Size of the amplified product** | **Primer names** | **Sequence (5’-3’)** | **Reference** |
| --- | --- | --- | --- | --- |
| PcPCL1606 | 378 bp | 04860F (forward) | CTTCGAATGGTCGGAACAAC | This study |
|  |  | 04860R (reverse) | GAATAGCAGCCTCGGTAAAC |  |
| *Rosellinia necatrix* | 493 bp | R2 (forward) | CAAAACCCATGTGAACATACCA | Schena et al., 2002 |
|  |  | R8 (reverse) | CCGAGGTCAACCTTTGGTATAG |  |
